# Neural responses to dynamic versus static food cues: A pilot functional MRI study

**DOI:** 10.1371/journal.pone.0361667

**Published:** 2026-10-28

**Authors:** Dabin Yeum, Susan J. Melhorn, Cindy Lao, Cole E. Anderson, Swati R. Levendovszky, Ellen A. Schur, Leticia E. Sewaybricker

**Affiliations:** 1 Department of Medicine, University of Washington, Seattle, Washington, United States of America; 2 Department of Radiology, University of Washington, Seattle, Washington, United States of America; Niigata University, JAPAN

## Abstract

Contemporary food environments are dominated by dynamic food cues such as moving videos of food, which may engage neural pathways differently than static images. Yet most neuroimaging studies of food cue reactivity have relied on static images. This preliminary study aimed to explore neural responses in reward- and appetite-regulating brain regions to dynamic versus static presentations of high- and low-calorie food stimuli. Seventeen healthy adults (8 with healthy weight and 9 with overweight; mean age = 32.7 ± 10.1y; 53% female) completed a novel functional magnetic resonance imaging (fMRI) paradigm involving dynamic (video) and static (image) presentations of food and non-food object stimuli. Two fMRI runs were conducted: high-calorie food and object cues were presented in Run 1 and low-calorie food and object cues were presented in Run 2. Stimulus format (dynamic vs. static) was manipulated within each run, and analyses of format effects were conducted separately for the high- and low-calorie conditions. Primary analyses examined whether stimulus format (dynamic vs. static) elicited differential activation of visual food cues relative to non-food objects, with high-calorie and low-calorie conditions tested separately. Regions of interest included *a priori* reward- and appetite-related regions, including the amygdala, dorsal striatum, ventral striatum, insula, and substantia nigra/ventral tegmental area. Secondary whole-brain analyses and exploratory comparisons by weight status were conducted. Stimulus format did not significantly modulate activation to food cues relative to objects in *a priori* reward- and appetite-related regions for either high- or low-calorie foods. Whole-brain analyses showed that the dynamic-versus-static high-calorie food contrast (relative to objects) elicited greater activation in the right occipital fusiform gyrus. Frontal, insular, occipital, and postcentral activation was observed to the dynamic high-calorie food-versus-object contrast. Exploratory analyses showed that, for high-calorie foods, individuals with overweight showed greater dynamic-versus-static food-related activation (relative to objects) in the dorsal striatum than individuals with healthy weight; for low-calorie foods, individuals with healthy weight showed greater dynamic-versus-static food-related activation (relative to objects) in the substantia nigra/ventral tegmental area than individuals with overweight. In this pilot study, effect sizes for dynamic-versus-static differences in reward-related regions did not reach statistical significance, and thus findings should be interpreted with caution given the limited sample size. Dynamic cues were well tolerated and showed similar appeal compared with static images, while engaging broader networks related to perception, attention, and salience. Larger studies and designs that randomize the food cue type are needed to confirm and extend these observations.

## Introduction

Food cues elicit distinct neural responses in the brain, with significant implications for understanding how individuals interact with food [1–3]. Static food cues, such as still images of food items, have been extensively studied [4,5] and are known to reliably engage brain regions associated with appetite regulation, reward, sensory processing, motivation, and decision-making, including the ventral striatum, amygdala, and insula [6–8]. Neural responses to food cues vary across individuals and have been linked to overeating and weight-related outcomes. Specifically, greater activation in reward-related regions and decreased inhibitory control have been observed in individuals with obesity or binge eating disorder, potentially contributing to excess caloric intake and dysregulated dietary regulation [9,10]. However, these static stimuli represent only a fraction of the complexity inherent in real-world food cue exposure. In contrast, dynamic food cues, such as moving videos of food being prepared, served, or consumed, are more temporally complex and may engage distinct reward pathways as they heighten our sensory experience of food beyond visual stimuli [11,12]. Prior work suggests that moving food stimuli recruit additional neural responses related to attention and sensory-motor integration, potentially engaging neural pathways that are not activated by static images alone [3,11] and may better represent naturalistic encounters with food.

The implementation of dynamic cues in research studies is well established in the broader cue reactivity literature regarding addiction. In the context of substance use disorders and behavioral addictions, dynamic cues, such as videos depicting drug use and tobacco use, have been shown to elicit stronger subjective craving and greater neural reactivity than static images, engaging regions involved in reward, attention, and motor preparation [13–16]. However, neuroimaging studies of food cue reactivity have largely relied on static image paradigms. Evaluating dynamic stimuli for visual food cues may better align with the contemporary food environment, which is increasingly dominated by video-based media, and may improve the capacity of experimental paradigms to assess food cue reactivity. In addition, there remains a need to establish and evaluate novel paradigms incorporating dynamic food cues, and to assess whether neural responses to dynamic food cues align with those demonstrated in the literature using static food cues.

In addition to stimulus properties, individual characteristics, such as body weight, may influence neural sensitivity to food cues. Evidence indicates that some individuals with overweight or obesity exhibit heightened neural responses to food-related stimuli, particularly in regions involved in reward processing, such as the striatum and insula [17–19]. This enhanced responsiveness may reflect inherited or acquired differences in food motivation or reward sensitivity, which, in turn, could influence behavioral phenotypes related to eating behavior that promote weight gain and impaired weight regulation. Whether dynamic food stimuli exacerbate or differentially modulate neural responsivity by weight status is an important question given the modern media landscape and deserves exploration.

The present study was designed as a pilot replication and extension of established static food-cue paradigms. Building on prior work using static food and object images [8, 20], we aimed to develop and validate a novel fMRI paradigm incorporating matching dynamic food videos alongside static images. The primary aim was to examine neural responses to dynamic versus static high-calorie food cues relative to non-food objects, using hypothesis-driven region-of-interest (ROI) analyses, complemented by whole-brain analysis as a secondary analysis. We hypothesized that dynamic high-calorie food cues would elicit greater activation in *a priori* reward- and appetite-related ROIs than static high-calorie food cues, using non-food object stimuli as a comparison condition to isolate food-specific responses. An exploratory aim was to examine whether neural responses to dynamic versus static food cues differed by weight status, recognizing the limited statistical power for definitive group comparisons.

## Materials and methods

### Study population and data collection

This cross-sectional pilot study included healthy adults aged 18–60 years old, able to undergo MRI, not pregnant/breastfeeding, and without major medical conditions. Twenty adults (10 with healthy weight (HW), BMI of 18.5–24.9 kg/m^2^, and 10 with overweight (OW), BMI of 25.0–29.9 kg/m^2^) were enrolled. Participants were instructed to fast for 4 hours prior to the study visit to reduce variability in appetitive state, consistent with prior food-cue neuroimaging studies [20]. Fasting was reinforced via reminder communications sent prior to the visit and verified by verbal self-report upon arrival. They underwent a single fMRI session with two runs of dynamic videos and static images: the first included high-calorie foods and objects, and the second included low-calorie foods and objects. Height and weight were measured using a stadiometer and a digital scale, respectively. Participants self-reported their biological sex. MRI data from three participants (N = 2 HW; N = 1 OW) were excluded due to inattention (e.g., falling asleep during the session). Participants were continuously monitored via the camera inside the scanner during scanning. Participants who exhibited sustained eye closure and lack of responsiveness to verbal prompts were classified as inattentive (e.g., falling asleep). Participants whose eyes remained closed for more than three consecutive blocks despite verbal prompts to remain awake were excluded from further analysis. This pilot was designed to evaluate feasibility and to obtain preliminary estimates of effect sizes for dynamic versus static food cue contrasts, which informed the sample size. This study was approved by the University of Washington Institutional Review Board on 23/02/2024. Recruitment for this study was conducted from February 2024 through August 2024, with the final participant visit on 28/08/2024. All subjects provided written informed consent.

### Stimuli development and validation

#### Static image stimuli.

Static food and object images were drawn from previously validated stimulus sets used in studies of neural responses to food cues [8,20,21]. Briefly, static images depicted three types of stimuli: 1) a ready-to-eat food item, either perceived as high-calorie (e.g., pastries, candy, pizza, hamburgers), or as 2) low-calorie (e.g., fruit, vegetables, salads, grilled chicken), and 3) objects. As done previously, foods were classified based on perceived energetic properties to identify cohesive stimulus sets with consistent nutritional and socio-cultural salience. High-calorie foods were those rated as unacceptable to eat when trying to lose weight and were typically high in calories, fat, and/or sugar (e.g., fries, desserts, and pizza). Low-calorie foods were those perceived as suitable to eat while dieting to lose weight. Object images included easily identifiable non-food related items (e.g., books, furniture, tools).

#### Dynamic video stimuli.

Dynamic stimuli consisted of commercial-quality stock videos obtained from video-sharing platforms or websites depicting foods or objects in motion. An initial pool of 71 food videos was assembled to closely match the static food images, with efforts to align the primary food or object components (e.g., an orange in both static and dynamic formats). All videos featured the food (or object) component as the focus, ready-to-eat items, and, in some images, people’s hands but not faces. Dynamic videos and static images were edited with the same frame size and display duration (2 seconds per stimulus) in Adobe Premiere Pro (version 15.0, 2021).

Independent raters (N = 47) evaluated each video using a validated rating approach adapted from prior static-image studies [8]. Raters were adults recruited independently from the imaging sample who provided their ratings anonymously; none participated in the fMRI study. To maintain consistency with prior image evaluation and selection protocols for studies of eating behavior and appetite regulation [8,22], raters responded to the prompt: *“If someone was dieting in order to lose weight, how acceptable would it be for them to eat this food?*” with the goal of identifying a set of videos that were 1) identified by the same means as the static images and 2) depicting foods widely perceived as highly caloric or low caloric. Videos with mean ratings ≤2 were classified as high-calorie, and those with ratings ≥6 were classified as low-calorie. Eleven videos (25.5%) were excluded due to ambiguous or mixed ingredient content (e.g., hamburger with side salad, multi-grain salad bowl, soup, fruit juice, or smoothie). The final stimulus set included 30 high-calorie food videos with a mean rating (SD) of 1.90 (1.21) and 30 low-calorie food videos with a mean rating (SD) of 6.63 (0.66). The final high-calorie food videos included cake being sliced, melted chocolate being poured over ice cream, and high-fat savory foods such as fries falling or a rotating burger. The final low-calorie food videos featured primarily fruits, vegetables, and lean proteins such as lettuce falling into a bowl of salad, rotating fruits, or a piece being cut from low-fat meat or seafood (e.g., chicken breast or fish). Non-food-related videos designated as object video group consisted of common, easily identifiable large and small objects such as toys, furniture, sports items, and sundries, and were free of any component related to eating (i.e., no utensils, straws, cups). All videos in the object condition depicted a single object shown in motion (e.g., a windmill spinning or a balloon floating).

All stimuli were matched for size (600 x 400 dpi), visual quality, and visual interest. To ensure comparability, mean luminosity was matched across all presentation formats, types, and blocks. A two-way ANOVA was conducted to assess differences in luminosity across stimulus types (high-calorie foods, low-calorie foods, and objects) and presentation formats (static image vs. dynamic video). The overall model was not significantly different (F(5, 246) = 0.60; *p* = .70); the interaction between type and format was not significant (F(2, 246) = 0.87; *p* = .42); the main effects of type and format were also not statistically significant (F(2, 246) = 0.52; *p* = .60 and F(1, 246) = 0.43; *p* = .51, respectively). Examples of final dynamic video stimuli for each condition (high-calorie foods, low-calorie foods, objects) are provided in S1 Video.

### Imaging paradigm

Participants underwent two functional runs during the fMRI session (Fig 1). The task was structured as two parallel 2 x 2 within-run designs, with cue type (food vs. object) and stimulus format (dynamic vs. static) manipulated within each run. High-calorie and low-calorie conditions were presented in separate runs and analyzed independently, rather than as a fully crossed factor. Run 1 included only high-calorie food and object stimuli and Run 2 included only low-calorie food and object stimuli. Each run consisted of 12 blocks: 6 food blocks (3 dynamic and 3 static) and 6 object blocks (3 static and 3 dynamic) (Fig 1). Each block presented 10 stimuli, either static images or dynamic videos, each image/video displayed for 2 seconds, resulting in 20 seconds per block. A 20-second fixation block with a gray screen was presented between each block to temporally separate conditions and allow the hemodynamic response to return to baseline; these periods were also modeled as the implicit baseline in the generalized linear model.

**Fig 1:**
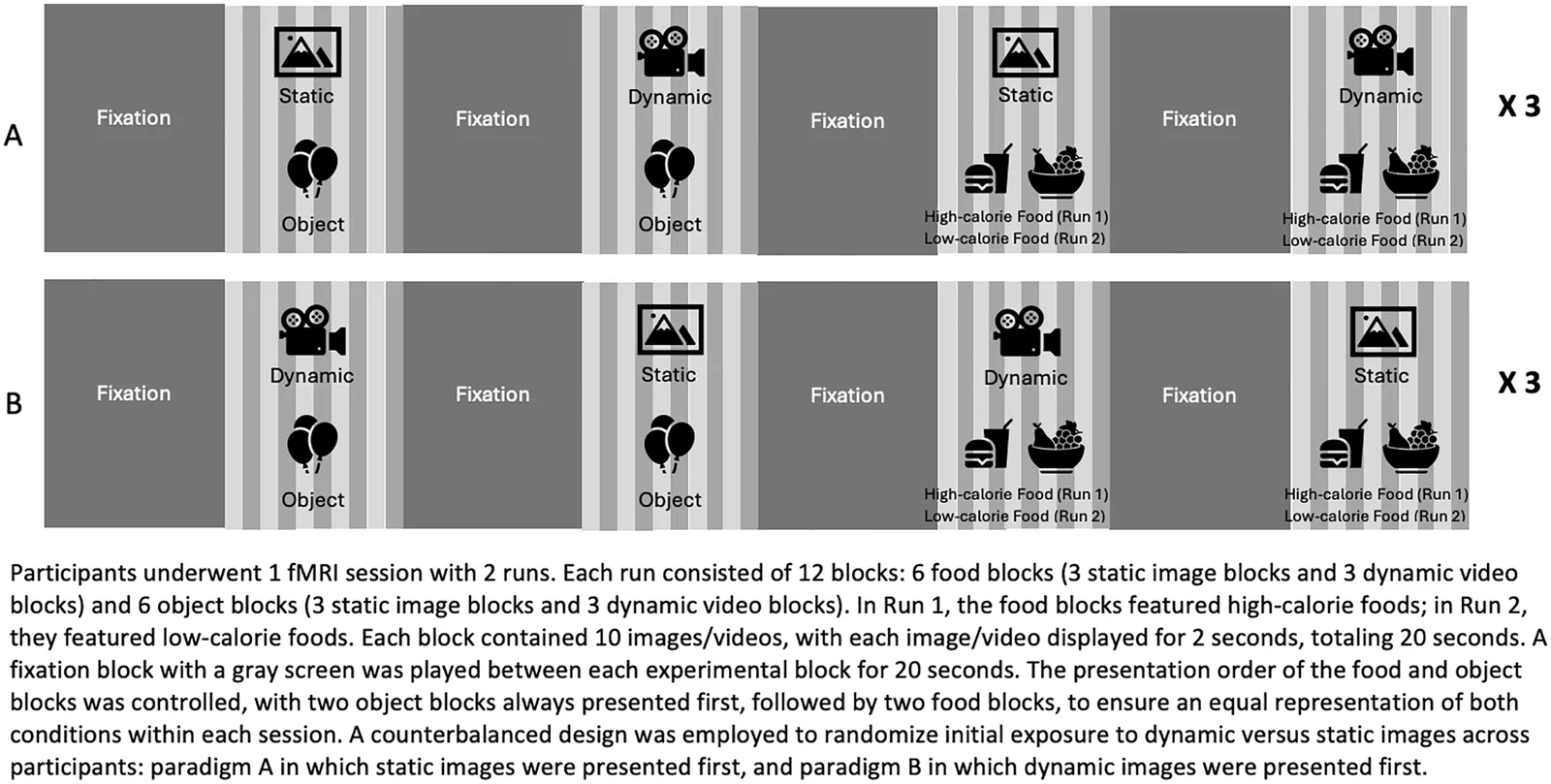
Summary of MRI paradigm. Participants underwent 1 fMRI session with 2 runs. The task followed a 2 (cue type: high-calorie or low-calorie food, object) X 2 (format: static images, dynamic videos) design. Each run consisted of 12 blocks: 6 food blocks (3 static image blocks and 3 dynamic video blocks) and 6 object blocks (3 static image blocks and 3 dynamic video blocks). In Run 1, the food blocks featured high-calorie foods; in Run 2, they featured low-calorie foods. Each block contained 10 images/videos, each displayed for 2 seconds, for a total of 20 seconds. A fixation block with a gray screen was played between each experimental block for 20 seconds. Block order was partially constrained, with object blocks presented before food blocks within each run. The presentation order of the food and object blocks was controlled, with two object blocks always presented first, followed by two food blocks, to ensure an equal representation of both conditions within each session. A counterbalanced design was employed to randomize initial exposure to dynamic versus static images across participants: paradigm A, in which static images were presented first, and paradigm B, in which dynamic images were presented first.

Block order was partially constrained rather than fully randomized. Paradigms were organized into block pairs matched by image type (food or object), with each pair consisting of one dynamic and one static block. Within each run, two object blocks (one dynamic, one static) were presented first, followed by two food blocks (one dynamic, one static). The order of dynamic and static blocks was counterbalanced across participants to account for order effects. That is, in paradigm A, participants viewed static images first; in paradigm B, dynamic videos were presented first. Stimuli were projected onto a screen inside the scanner and viewed through a mirror mounted on the head coil. Participants were instructed to think about how the food would taste or how the object would feel to touch.

After the fMRI session, a post-MRI memory task was administered outside of the scanner, consisting of 32 images: 16 previously viewed (8 high-calorie foods and 8 low-calorie foods; equally divided between dynamic videos and static images) and 16 novel distractors. Each distractor was matched to a previously viewed item by specific food type (e.g., a previously viewed image of pizza was paired with a novel image of a different pizza). Participants were asked to indicate whether they had seen each image during the scan, and the percentage of correct responses was calculated as a descriptive measure of attention and engagement; no additional participants were excluded based on this task.

In addition, participants completed an appeal rating questionnaire in which they viewed 24 food stimuli previously shown during the scan (6 high-calorie dynamic videos, 6 high-calorie static images, 6 low-calorie dynamic videos, and 6 low-calorie static images). For each stimulus, participants were asked to rate how much they wanted to eat the food at that moment, using a Likert scale from 1 (not appealing at all) to 10 (extremely appealing). Ratings were used to assess subjective food appeal across food types (high-calorie vs. low-calorie) and presentation formats (static vs. dynamic).

### Image acquisition and processing

Scans were acquired with a 32-channel, SENSE head coil on a 3-T Philips Ingenia Elition System (Software Version 5.7.1, Philips Medical Systems, Netherlands) with 45mT/m gradient strength and 220mT/m/s slew rate. Echo-planar imaging time-series (51 axial slices, 2.5 mm isotropic voxels, TR = 1000ms, TE = 25ms, Flip angle = 70°, SENSE factor = 1.7, simultaneous multi-slice (SMS) factor = 3) was acquired during passive image/video viewing. To correct for distortions, two spin echo acquisitions (opposite phase-encoding, no SMS settings, TR = 5000ms, TE = 60ms) were performed. The imaging plane was tilted 18° relative to the AC-PC line to reduce the inclusion of the orbits and, consequently, minimize signal dropout in the frontal regions caused by susceptibility artifacts. The FSL’s top-up algorithm with two reversed phase-encoded images was applied for distortion correction [23].

Anatomical scans were collected using a 3D Compressed Sense Magnetization-Prepared Rapid Gradient-Echo (MPRAGE) image and registered the functional data to standard space. MPRAGE and functional image processing procedures were previously published [7,21]. Briefly, anatomical scans were skull-stripped and aligned to the MNI standard space. Functional preprocessing included slice timing correction, motion correction, spatial smoothing (5 mm FWHM), and high-pass temporal filtering. Functional images were registered to the individual’s structural scan and to standard space using non-linear warping. First-level models were implemented using a general linear model (GLM) in FEAT (FMRI Expert Analysis Tool) to define subject-level contrasts for each condition of interest. Preprocessing and first-level analyses were conducted using FEAT within the FSL software package [23,24]. Functional runs with excessive motion, defined as a mean framewise displacement greater than 1 mm and more than 15% of total volumes identified as outliers, were flagged for potential exclusion; however, no participants met these criteria. Additional quality control steps included visual inspection of anatomical-functional registration.

### Statistical modeling and analysis

The primary analyses focused on *a priori* ROI comparisons, selected to replicate and extend prior static food-cue neuroimaging findings [7,20]. These ROIs included substantia nigra/ventral tegmental area (SN/VTA) and bilateral regions of the amygdala, dorsal striatum, ventral striatum, and insula; regions consistently defined and shown to be markers of satiety or predictors of food choice in paradigms involving viewing static high-calorie food images [7, 20]. Briefly, ROIs were defined using a combined anatomical and functional approach. Anatomical masks from the Harvard-Oxford probabilistic atlas included the bilateral amygdala, dorsal striatum, ventral striatum, and insula. Then, functional ROIs were created by identifying voxels within the anatomical ROIs that exhibited significantly greater blood-oxygen-level-dependent (BOLD) responses to food stimuli compared to object stimuli (*p* < 0.05, uncorrected), collapsed across both dynamic and static formats using a mixed-effects model. Additionally, the SN/VTA mask was anatomically defined based on midbrain landmarks [7].

Condition effects were estimated separately for the high-calorie (Run 1) and low-calorie (Run 2) stimuli. Within each run, neural responses were modeled at the subject-level using separate regressors for dynamic foods (DF), static foods (SF), dynamic objects (DO), and static objects (SO), each contrasted against fixation, consistent with standard practice for ROI-based analyses. First-level models were estimated using a GLM in FEAT within FSL (version 6.0). For the primary analysis, neural sensitivity to stimulus format was examined by contrasting dynamic versus static activation to high-calorie foods relative to object cues (Run 1) and low-calorie foods relative to object cues (Run 2), analyzed separately. For each participant, mean parameter estimates for this contrast were extracted for each *a priori* ROI using FSL’s fslmaths and fslstats. Paired t-tests were conducted in R (version 4.3.1; R Core Team, 2023 to compare mean parameter estimates between dynamic and static food-related contrast (relative to object cues), examined separately for each *a priori* ROI and each calorie cue condition. Effect sizes were calculated as Cohen’s *d* for paired comparisons, computed as the mean differences between dynamic and static conditions divided by the standard deviations of the differences. Analyses were conducted at the ROI level to characterize region-specific activation patterns within a hypothesis-driven framework, rather than voxel-wise inference. Secondary pairwise ROI comparisons (e.g., DF > SF, DO>SO, DF > DO contrasts estimated separately for high-calorie and low-calorie conditions) are reported descriptively to aid interpretation of the primary dynamic vs. static contrast and to replicate the prior findings of neural activation to food images in the static condition (e.g., static high-calorie food cues relative to objects and static low-calorie food cues relative to objects). ROI-based analyses focused on *a priori*, theory-driven regions implicated in reward and appetite regulation. Given these predefined hypotheses and the limited sample size of this pilot study, the results were interpreted with caution and are intended to inform replication in larger samples.

To complement the ROI analyses and to explore the anatomic locations of voxel-wise activation in an untargeted analysis, secondary whole-brain analyses were conducted using FLAME1 mixed-effects models in FSL and were examined separately for high-calorie (Run 1) and low-calorie (Run 2) stimuli. Exploratory whole-brain analyses were conducted using a voxel-wise threshold of p < 0.01, combined with a cluster-extent threshold of p < 0.05, and further controlled for multiple comparisons using false discovery rate (FDR) correction at p < 0.05.

Finally, to explore potential effect modification by weight status, post hoc subgroup analyses compared neural activation between participants with healthy weight and overweight.

## Results

Of all enrolled participants, 20 completed the full study visit and MRI protocol. However, three participants (2 with HW; 1 with OW) were excluded from analysis due to inattention during the scan (e.g., falling asleep), resulting in an analytic sample of N = 17. Among participants included in the analysis (N = 17, 53% female), mean(SD) age was 32.7(10.1) years and BMI was 25.1(3.0) kg/m^2^. Participants (N = 17) correctly identified previously seen images and videos with a mean accuracy on the post-MRI memory task of 78% (SD = 13%), reflecting adequate attention and engagement during the scan. Among participants included in the analysis (N = 17), appeal ratings of dynamic high-calorie, dynamic low-calorie, static high-calorie, and static low-calorie food cues were high overall, ranging from 5.90–6.75 (out of 10) (S2 Table). Appeal ratings were examined separately within each calorie condition and did not differ significantly by stimulus format (static vs. dynamic) or by types of food cues (high vs. low-calorie) for the stimuli presented during the MRI session. Weight categories were defined as participants with HW (N = 8) or OW (N = 9) (22.6 vs 27.4 kg/m^2^, 29.4 vs 35.7 years, respectively).

Pre-scan hunger ratings were moderately high (mean = 56.6, SD = 23.6 on a 1–100 scale), while pre-scan fullness ratings were relatively low (mean = 21.1, SD = 23.9), indicating variability in appetitive state at the start of the scan. Pre-scan hunger and fullness ratings did not differ significantly between individuals with healthy weight and overweight (all *p*’s > 0.05; S3 Table), suggesting that the appetitive state was comparable between weight groups at the time of scan. Post scan feedback was generally positive. No participants reported discomfort, nausea, or distress related to the paradigm, indicating good tolerability. The most commonly reported concern was sleepiness during fixation blocks, consistent with previous concerns regarding the passive viewing nature of the task in the confined MRI environment. No runs were terminated early.

Within the selected *a priori* ROIs, no significant differences were observed between dynamic and static food cues when contrasted with object responses. In Run 1 (high-calorie foods), dynamic and static activation to high-calorie foods (relative to objects) did not reveal significant differences in activation within any of the *a priori* ROIs (all *p*’s > 0.05; Table 1). Similarly, in Run 2 (low-calorie foods), no significant differences were observed between dynamic and static activation to low-calorie foods (relative to objects) within any of the *a priori* ROIs (all *p*’s > 0.05; S4 Table). Observed effect sizes for the dynamic versus static food-related contrast were negligible to small for both high-calorie (d = −0.11 to 0.46) and low-calorie (d = −0.09 to 0.31) conditions, with the dorsal striatum showing the largest effects in both calorie conditions (Table 1 and S4 Table). Together, these findings suggest that stimulus format (dynamic vs. static) did not robustly modify food-related neural responses in reward- and appetite-related ROIs across the two calorie conditions.

**Table 1:** Region-of-interest comparisons of dynamic versus static format of high-calorie food (vs. Object) Cues (N = 17).

Regions-of-interest	Dynamic (high-calorie food>object) β Mean (SD)	Static (high-calorie food>object) β Mean (SD)	*t-value*	*p-value*	*Cohen’s d*
Amygdala	13.1 (47.2)	18.6 (50.7)	−0.47	.642	−0.11
Dorsal Striatum	22.9 (49.8)	5.67 (19.2)	1.26	.226	0.46
Ventral Striatum	−1.11 (46.9)	−1.44 (32.5)	0.02	.981	0.01
Insula	21.0 (39.0)	19.7 (44.3)	0.10	.924	0.03
SN/VTA^b^	24.2 (37.0)	27.2 (53.7)	−0.21	.838	−0.06

Values represent extracted mean parameter estimates (SD) for the high-calorie food>object contrast, averaged across bilateral regions. *t*-values, *p*-values, and Cohen’s d are from paired *t*-tests comparing dynamic and static stimulus formats. ^a^SN/VTA = Substantia Nigra/Ventral Tegmental Area.

In secondary whole-brain analyses, dynamic versus static activation to high-calorie foods (relative to objects) elicited greater responses in the right occipital fusiform gyrus (FDR-corrected *p* < 0.001; Table 2). Additional contrasts showed greater activation for dynamic high-calorie foods compared to dynamic objects in regions including the right occipital pole, left frontal orbital cortex, right postcentral gyrus, and right insular cortex (all FDR-corrected *p*’s < 0.004). Static high-calorie foods elicited greater activation than static objects in the left occipital pole (*p* = 0.010). Direct comparisons of dynamic versus static food stimuli showed greater activation in the left lateral occipital cortex for dynamic foods (*p* < 0.001). For object stimuli, dynamic cues elicited greater activation than static cues in bilateral lateral occipital and superior parietal regions (all FDR-corrected *p*’s < 0.011), while static object cues showed greater activation than dynamic cues in occipital, temporal, and motor regions, including the right juxtapositional lobule cortex and right planum polare (all FDR-corrected *p*’s < 0.04). No significant clusters were observed in the static versus dynamic high-calorie food contrast. In Run 2 (low-calorie foods), no clusters showed significant differences for the dynamic versus static activation to low-calorie foods (relative to objects) (S5 Table). However, dynamic low-calorie foods elicited greater activation than dynamic objects across multiple regions, including the lingual gyrus, lateral occipital cortex, frontal pole, cingulate gyrus, and insular cortex (all FDR-corrected *p*’s<0.022). Static low-calorie foods also showed greater activation than static objects in the occipital pole, frontal orbital cortex, and cingulate gyrus. Finally, comparisons between dynamic and static low-calorie food stimuli showed greater activation in the left middle frontal gyrus for dynamic foods (*p* < 0.001).

**Table 2:** Areas showing differential activation by image type (high-calorie food vs. object) and format (dynamic vs. static).

Region(s)	Cluster Size (voxels)	Peak Z value	Peak Coordinates			P (FDR-corr)
			X	Y	Z	
Run 1 (high-calorie foods)						
**Dynamic vs. Static (high-cal food>object)**						
Right Occipital Fusiform Gyrus	2859	3.93	28	−78	−16	<0.001
**Dynamic high-cal food > Dynamic object**						
Right Occipital Pole	7869	4.67	12	−94	−2	<0.001
Left Frontal Orbital Cortex	1237	4.72	−24	34	−12	<0.001
Right Postcentral Gyrus	1090	3.94	62	−18	30	<0.001
Right Insular Cortex	694	3.97	40	4	−12	0.005
**Static high-cal food > Static object**						
Left Occipital Pole	573	3.88	−20	−100	−2	0.010
**Dynamic high-cal food>** **Static high-cal food**						
Left Lateral Occipital Cortex	10519	5.24	−44	−68	16	<0.001
**Dynamic object > Static object**						
Right Lateral Occipital Cortex, inferior div.	2278	4.89	46	−70	0	<0.001
Left Lateral Occipital Cortex, inferior div.	1535	5.12	−48	−70	10	<0.001
Left Lateral Occipital Cortex, superior div.	916	5.4	−16	−86	34	<0.001
Right Superior Parietal Lobule	840	4.32	28	−50	64	<0.001
Right Lateral Occipital Cortex	681	4.99	20	−86	36	0.003
Left Superior Parietal Lobule	530	4.56	−34	−46	60	0.011
**Static vs. Dynamic (high-cal food>object)**						
*NA*						
**Dynamic object> Dynamic high-cal food**						
Right Lateral Occipital Cortex, superior div.	943	4.07	44	−70	16	<0.001
**Static object> Static high-cal food**						
Left Lateral Occipital Cortex, superior div.	989	4.31	−42	−78	34	<0.001
Right Lateral Occipital Cortex, superior div.	539	3.67	44	−82	22	0.015
**Static high-cal food>** **Dynamic high-cal food**						
*NA*						
**Static object > Dynamic object**						
Right Occipital Pole	3253	4.06	14	−94	−2	<0.001
Right Juxtapositional Lobule Cortex	1461	4.01	4	0	74	<0.001
Right Planum Polare	769	3.59	56	6	0	0.001
Left Superior Temporal Gyrus, posterior div.	727	3.71	−62	−34	6	0.002
Left Temporal Fusiform Cortex, posterior div.	422	3.29	−36	−20	−18	0.04

Exploratory analyses examined whether neural responses to dynamic versus static activation to high-calorie or low-calorie foods (relative to objects) differed by weight status across *a priori* reward- and appetite-related ROIs. In Run 1 (high-calorie foods), participants with OW (β Mean = 45.8, SD = 49.2) showed greater activation in the dorsal striatum for dynamic versus static high-calorie food cues, relative to object cues, compared with participants with HW (β Mean = −15.0, SD = 47.2; *t*(15) = 2.59; *p* = .020, *Cohen’s d* = 1.26, uncorrected; S6 Table). No significant weight-status differences were observed in the amygdala, ventral striatum, insula, or SN/VTA (all *p*’s>0.05). In Run 2 (low-calorie foods), a significant weight-status difference was observed in the substantia nigra/ventral tegmental area, with greater dynamic versus static activation to low-calorie foods (relative to objects) in those with HW relative to those with OW (*t* = −3.12; *p* = .008, *Cohen’s d* = −1.52, uncorrec*t*ed), with no other ROIs reaching statistical significance. These exploratory comparisons were not corrected for multiple comparisons.

## Discussion

The present study introduces a novel fMRI paradigm that uses dynamic food videos to assess neural responses to food cues across brain regions involved in reward and appetite processing. This approach addresses a critical gap in the literature, as most prior neuroimaging studies of food cue reactivity have relied on static images [25, 26] despite the predominance of dynamic food content in contemporary media environments. Importantly, the paradigm was feasible, as all 20 enrolled participants completed the full study visit and MRI protocol; however, three participants were excluded from the analysis due to inattention during the scan. Engagement during the task was supported by post-MRI memory task accuracy, and tolerability was supported by post-scan feedback indicating no reports of discomfort, nausea, or distress and no early termination of MRI runs. Together, these findings support the feasibility and tolerability of the paradigm in this pilot sample while highlighting considerations for optimizing participant engagement for future, larger-scale investigations.

Contrary to our primary hypothesis, dynamic versus static food cues did not elicit statistically significant differences in activation within *a priori* reward-related ROIs when contrasted with object responses for either high- or low-calorie food conditions. However, these findings should be interpreted with caution, given the limited statistical power of this pilot study. Observed effect sizes were negligible to small across most ROIs, suggesting that format-related differences in reward-related neural responses to food cues may be modest on average across all participants. Notably, the broadly similar pattern of activation across dynamic and static food cues suggests that findings from the extensive literature on static food images may remain relevant when interpreting studies that employ more ecologically valid dynamic stimuli. This is encouraging given that one of the primary goals of the present study was to establish the feasibility and tolerability of a dynamic food cue paradigm that more closely reflects food-related media encountered in contemporary environments. Additionally, exploratory analyses identified weight-status differences in neural responses to dynamic versus static food cues in the dorsal striatum for high-calorie foods and in the substantia nigra/ventral tegmental area for low-calorie foods, suggesting that stimulus format may be more relevant in specific subpopulations than in the overall sample. Power analyses based on these observed effect sizes in the dorsal striatum for high-calorie foods (d = 0.46), using a paired t-test at α = 0.05 with 80% power, indicate that approximately 40 participants would be required to detect effects of this magnitude. Together, these findings suggest that format-related effects are modest compared to the overall consistently demonstrated robust responsiveness to visual food stimuli and that preliminary effect size estimates support both the use of dynamic stimuli and future studies of potential subgroup-specific differences.

Beyond statistical power, other factors may contribute to the absence of robust format effects in reward-related regions. In the addiction literature, similar null or mixed findings have been reported in other cue-reactivity domains, including substance use, where dynamic cues do not consistently elicit stronger reward-related responses than static cues, and where individual-level factors, such as personal relevance or learning history, may play a more important role than cue format [27,28]. These studies suggest that the salience or personal relevance of the cue depends less on its presentation format and more on factors such as current hunger state, dietary restraint, or prior exposure to specific foods. These findings also suggest that incorporating additional sensory modalities, such as olfactory cues, could capture the multisensory nature of real-world food cue exposure and its effect on reward-related neural processing.

Secondary whole-brain analyses showed that the dynamic versus static high-calorie food (relative to object) cues were associated with greater activation in the occipital regions, including the fusiform gyrus, a region implicated in high-level visual processing, such as object recognition and category-specific representations [29]. In contrast, no significant dynamic-versus-static differences were observed for the low-calorie food cues. Comparisons of dynamic high-calorie food cues relative to object stimuli showed activation in frontal, insular, and occipital, and postcentral regions implicated in attentional and salience-related processing [30,31]. Notably, dynamic-versus-static differences were observed for both food and object stimuli in occipital and parietal regions, indicating that dynamic presentation enhances visual and attentional processing associated across stimulus types. These findings suggest that dynamic presentation may enhance perceptual and attentional engagement, particularly for high-calorie foods.

Exploratory analyses further suggested that weight status may modify neural sensitivity to stimulus format in a region- and calorie-specific manner. For high-calorie foods, individuals with overweight exhibited greater dynamic-versus-static food-related activation in the dorsal striatum relative to those with healthy weight. The dorsal striatum has been linked to stimulus-response and habit learning as well as action selection in instrumental behavior rather than hedonic reward processing [32,33], suggesting the possibility that dynamic high-calorie food cues may preferentially engage habit-based and action-selection neural pathways in this group. In contrast, for low-calorie foods, the substantia nigra/ventral tegmental area showed a weight-status difference with greater dynamic-versus-static activation in participants with healthy weight. These preliminary findings suggest that dynamic presentation may differentially engage neural processing depending on stimulus content, brain region, and individual characteristics, and warrant replication in larger, adequately powered studies.

This study has several important limitations. First, the small sample size limits statistical power, increasing the risk of both Type I and Type II errors. ROI analyses were restricted to *a priori*, theory-driven regions and were therefore not adjusted for multiple comparisons across ROIs. Although functional ROIs were derived from the same dataset, circularity was minimized by collapsing across all food categories and stimulus formats to define the ROI mask, thereby identifying a minimal level of responsiveness to food cues while avoiding the introduction of contrast-specific bias into the primary dynamic-versus-static analysis. Due to a small sample size (N = 8 vs. N = 9), group comparisons were particularly underpowered for smaller effect sizes, with fewer than 10 participants per group, limiting power to detect true group differences. Although prespecified as an exploratory analysis, these findings should be interpreted strictly as hypothesis-generating and were not corrected for multiple comparisons. Second, the study did not include participants with obesity, limiting generalizability. As individuals with obesity or binge eating disorder may show heightened reward responsivity, altered inhibitory control, and greater cue-induced craving in response to food cues [9–11], the present paradigm may capture only a subset of the variability in the neural reactivity that is most clinically relevant for vulnerable populations at elevated risk for overeating. Future studies should assess individuals with obesity and/or eating disorders to examine whether dynamic cue format exacerbates food cue reactivity and whether such responses are associated with clinically meaningful behavioral outcomes. Third, neural activation to food cues was not paired with behavioral or physiological indices of appetite or consumption, such as *ad libitum* food intake or hormonal measures. Although appeal ratings did not differ by stimulus type, this may reflect limited power or social desirability bias. Fourth, although a standardized 4-hour fasting period was implemented to reduce variability in appetitive state, this approach may not fully equalize hunger across participants given individual differences in metabolism and meal composition. However, pre-scan hunger and fullness rating did not differ between weight groups. Dynamic food videos may also elicit stronger cephalic phase responses, including anticipatory physiological changes, which were not measured. Fifth, while stimulus presentation order was counterbalanced, order effects (e.g., novelty associated with viewing dynamic videos first) were not modeled as covariates. While this design was implemented to prioritize comparisons of dynamic versus static stimulus format within each run, it may introduce potential order effects, particularly related to the fixed sequencing of dynamic and static blocks and the presentation of high-calorie stimuli in Run 1 and low-calorie stimuli in Run 2, as high-calorie food cues represent the primary condition of interest and low-calorie food cues as a secondary condition. Although fixation blocks were included between stimulus blocks to minimize carryover effects, residual influences of prior stimulus exposure may still be present. Future studies should incorporate full randomization of cue type and presentation format to isolate these effects. Lastly, the 2-second presentation duration for dynamic video stimuli was chosen deliberately to match the static image duration, ensuring experimental control and comparability across formats. Furthermore, this brief duration mirrors the short-form media clips and GIFs in the digital food environments. While this brief exposure minimizes potential habituation from rapid serial stimulus transitions during the MRI scan, it may have limited the depth to which the full dynamic nature of certain cues could be integrated. Future studies should systematically evaluate how varying presentation durations for dynamic food cue paradigms impact neural responses.

Despite these limitations, the present study introduces a dynamic food-cue paradigm that incorporates stimulus formats more representative of contemporary food environments, providing a foundation for future investigations into how food-cue format might influence neurocognitive and reward processing. These findings suggest that dynamic food cues engage broader networks related to perception, attention, and salience, highlighting the potential impact of dynamic food cues, such as those commonly found in marketing and media, on neural reward processing in at-risk populations. However, the extent to which this broader neural engagement translates into greater ecological validity or stronger cue-induced appetitive responses remains to be established. In sum, adapting investigations of eating behavior to reflect the contemporary food marketing environment is critical to advancing our understanding of the impact of such cue exposures on appetite, body weight regulation, and ultimately, health.

## Supporting information

S1 Video. Examples of dynamic video stimuli for each condition.
(DOCX)

S2 Table Appeal ratings by food type and presentation format (N = 17). (DOCX)

S3 Table Visual analog scale (VAS) hunger and fullness ratings by weight status (N = 17). (DOCX)

S4 Table Region-of-Interest Comparisons of Dynamic Versus Static Format of Low-calorie Food Cues (food>object) (N = 17). (DOCX)

S5 Table Areas showing differential activation by image type (low-calorie food vs. object) and format (dynamic vs. static). (DOCX)

S6 Table Differences in Brain Activation by Dynamic (high-calorie food>object) Versus Static (high-calorie food>object) Cues by weight status (overweight [N = 9] vs. healthy weight [N = 8]). (DOCX)

## Abbreviations

fMRI: functional magnetic resonance imaging
ROI: region of interest
HW: healthy weight
OW: overweight
CI: confidence interval
