# Supplementary material for: Neural responses to dynamic versus static food cues: A pilot functional MRI study
Source: PLoS One. 2026 Oct 28;21(10):e0361667. doi: 10.1371/journal.pone.0361667 (PMC13716230; doi:10.1371/journal.pone.0361667)
Supplement: Supplementary file 1 [file pone.0361667.s001.docx]

**Supp Video 1. Examples of dynamic video stimuli for each condition.**

| High-calorie food |  |
| --- | --- |
| Low-calorie food |  |
| Object |  |
