# Supplementary material for: Neural responses to dynamic versus static food cues: A pilot functional MRI study
Source: PLoS One. 2026 Oct 28;21(10):e0361667. doi: 10.1371/journal.pone.0361667 (PMC13716230; doi:10.1371/journal.pone.0361667)
Supplement: Supplementary file 2 [file pone.0361667.s002.docx]

**Supp Table 2. Appeal ratings by food type and presentation format (N=17).**

| Comparison | Condition 1  Mean (SD) | Condition 2  Mean (SD) | *t-value* | *p-value* |
| --- | --- | --- | --- | --- |
| Dynamic high-calorie > Static high-calorie | 6.62 (2.19) | 6.75 (2.35) | -0.18 | .862 |
| Dynamic low-calorie > Static low-calorie | 6.26 (1.85) | 5.90 (1.49) | 0.63 | .534 |

Values represent mean appeal ratings per stimulus on a 1-10 Likert scale (1 = not appealing at all, 10 = extremely appealing), averaged across 6 stimuli per condition. Comparisons were conducted using Welch two-sample t-tests.
