# Supplementary material for: Neural responses to dynamic versus static food cues: A pilot functional MRI study
Source: PLoS One. 2026 Oct 28;21(10):e0361667. doi: 10.1371/journal.pone.0361667 (PMC13716230; doi:10.1371/journal.pone.0361667)
Supplement: Supplementary file 3 [file pone.0361667.s003.docx]

**Supp Table 3. Visual analog scale (VAS) hunger and fullness ratings by weight status (N=17).**

| Comparison | Overall  Mean (SD) | HW  Mean (SD) | OW  Mean (SD) | *t-value* | *p-value* |
| --- | --- | --- | --- | --- | --- |
| Pre-scan hunger | 56.6 (23.6) | 61.9 (16.1) | 52.0 (28.9) | 0.88 | 0.394 |
| Pre-scan fullness | 21.2 (23.9) | 15.1 (19.0) | 26.7 (27.5) | -1.01 | 0.327 |
| Post-scan hunger | 75.1 (10.4) | 71.8 (8.0) | 78.0 (11.8) | -1.29 | 0.216 |
| Post-scan fullness | 15.4 (14.4) | 13.6 (12.8) | 17.0 (15.8) | -0.49 | 0.634 |
| Hunger changes (post-pre) | 18.4 (18.3) | 9.9 (12.9) | 26.0 (11.9) | -2.02 | 0.063 |
| Fullness changes (post-pre) | -5.8 (14.7) | -1.5 (19.6) | -9.7 (16.5) | 1.18 | 0.257 |

VAS ratings were collected on a 0-100 scale. Hunger and fullness change scores reflect post-scan minus pre-scan ratings. Group comparisons of HW and OW were conducted using two-sample t-tests.

HW = healthy weight

OW = overweight
