# Supplementary material for: Neural responses to dynamic versus static food cues: A pilot functional MRI study
Source: PLoS One. 2026 Oct 28;21(10):e0361667. doi: 10.1371/journal.pone.0361667 (PMC13716230; doi:10.1371/journal.pone.0361667)
Supplement: Supplementary file 4 [file pone.0361667.s004.docx]

**Supp Table 4. Region-of-Interest Comparisons of Dynamic Versus Static Format of Low-calorie Food (vs. Object) Cues (N=17).**

| Regions-of-interest | Dynamic(low-calorie food>object) β  Mean (SD) | Static(low-calorie food>object) β  Mean (SD) | *t-value* | *p-value* | *Cohen’s d* |
| --- | --- | --- | --- | --- | --- |
| Amygdala | 16.9 (47.4) | 17.9 (37.7) | -0.08 | .940 | .024 |
| Dorsal Striatum | 16.6 (33.6) | 5.54 (38.9) | 0.97 | .344 | .305 |
| Ventral Striatum | 32.9 (50.5) | 25.9 (49.3) | 0.40 | .692 | .141 |
| Insula | 23.9 (40.8) | 27.5 (43.9) | -0.24 | .814 | -.085 |
| SN/VTA^a^ | 18.5 (38.5) | 11.2 (40.0) | 0.56 | .581 | .188 |

Values represent extracted mean parameter estimates for the low-calorie food>object contrast, averaged across bilateral regions. *t*-values, *p*-values, and Cohen’s d are from paired t-tests comparing dynamic and static stimulus formats.

^a^ SN/VTA = Substantia Nigra/Ventral Tegmental Area
