# Supplementary material for: Neural responses to dynamic versus static food cues: A pilot functional MRI study
Source: PLoS One. 2026 Oct 28;21(10):e0361667. doi: 10.1371/journal.pone.0361667 (PMC13716230; doi:10.1371/journal.pone.0361667)
Supplement: Supplementary file 5 [file pone.0361667.s005.docx]

**Supp Table 5. Anatomical brain regions showing differential activation by image type (low-calorie food vs. object) and format (dynamic vs. static)**

| Region(s) | Cluster Size (voxels) | Peak Z value | Peak Coordinates | | | P (FDR-corr) |
| --- | --- | --- | --- | --- | --- | --- |
|  |  |  | X | Y | Z |  |
| *Run 2 (low-calorie foods)* |  |  |  |  |  |  |
| **Dynamic vs. Static (low-calorie food>object)** |  |  |  |  |  |  |
| N/A |  |  |  |  |  |  |
| **Dynamic low-calorie food > Dynamic object** |  |  |  |  |  |  |
| Left Lingual Gyrus | 3939 | 4.62 | -8 | -88 | -10 | <0.001 |
| Right Frontal Pole | 1164 | 3.78 | 42 | 54 | -2 | <0.001 |
| Right Lateral Occipital Cortex | 1054 | 4.57 | 42 | -68 | 48 | <0.001 |
| Cingulate Gyrus | 999 | 4.1 | 0 | -30 | 30 | <0.001 |
| Right Insular Cortex | 819 | 3.85 | 36 | 10 | -8 | 0.001 |
| Left Lateral Occipital Cortex | 507 | 3.3 | -50 | -62 | 40 | 0.022 |
| **Static low-calorie food > Static object** |  |  |  |  |  |  |
| Right Occipital Pole | 4617 | 4.98 | 16 | -90 | 0 | <0.001 |
| Right Frontal Orbital Cortex | 1889 | 4.46 | 24 | 34 | -14 | <0.001 |
| Right Cingulate Gyrus | 786 | 3.95 | 4 | -38 | 36 | 0.003 |
| **Dynamic low-calorie food >**  **Static low-calorie food** |  |  |  |  |  |  |
| Left Middle Frontal Gyrus | 937 | 3.96 | -30 | 8 | 60 | <0.001 |

All voxel-wise p<0.01 uncorrected and cluster-level p<0.05
