# Supplementary material for: Neural responses to dynamic versus static food cues: A pilot functional MRI study
Source: PLoS One. 2026 Oct 28;21(10):e0361667. doi: 10.1371/journal.pone.0361667 (PMC13716230; doi:10.1371/journal.pone.0361667)
Supplement: Supplementary file 6 [file pone.0361667.s006.docx]

**Supp Table 6. Differences in Brain Activation by Dynamic (high-calorie food>object) Versus Static (high-calorie food>object) Cues by weight status (overweight [N=9] vs. healthy weight [N=8])**

| Dependent Variable | Overweight β  Mean (SD) | Healthy weight β  Mean (SD) | *t-value* | *p-value* | *Cohen’s d* |  |
| --- | --- | --- | --- | --- | --- | --- |
| **Dynamic vs. Static (high-calorie food > object)** | | |  |  |  |  |
| Amygdala | 13.1 (50.8) | -26.6 (38.1) | 1.83 | 0.087 | 0.88 |  |
| Dorsal Striatum | **45.8 (49.2)** | **-15.0 (47.2)** | **2.60** | **0.020*** | **1.26** |  |
| Ventral Striatum | 17.7 (52.9) | -19.2 (56.8) | 1.38 | 0.188 | 0.67 |  |
| Insula | 11.2 (62.4) | -9.85 (48.9) | 0.78 | 0.448 | 0.37 |  |
| Substantia Nigra/  Ventral Tegmental Area | 0.607 (67.6) | -7.04 (53.1) | 0.26 | 0.798 | 0.12 |  |
| **Dynamic vs. Static (low-calorie food > object)** | | |  |  |  |  |
| Amygdala | 5.97 (62.2) | -8.89 (48.2) | 0.55 | 0.588 | 0.26 |  |
| Dorsal Striatum | -4.88 (52.9) | 29.1 (33.8) | -1.59 | 0.134 | -0.75 |  |
| Ventral Striatum | -20.8 (61.7) | 38.4 (73.0) | -1.79 | 0.095 | -0.88 |  |
| Insula | -26.6 (67.9) | 22.3 (46.6) | -1.74 | 0.103 | -0.83 |  |
| Substantia Nigra/  Ventral Tegmental Area | **-23.6 (39.8)** | **42.3 (47.2)** | **-3.09** | **0.008*** | -1.52 |  |

Values represent extracted mean parameter estimates for the food>object for each weight category. *t*-values and *p*-values are from t-tests comparing participants with overweight and healthy weight. Healthy weight category was used as the reference group.

* p<0.05
