# Supplementary material for: Neural responses to dynamic versus static food cues: A pilot functional MRI study
Source: PLoS One. 2026 Oct 28;21(10):e0361667. doi: 10.1371/journal.pone.0361667 (PMC13716230; doi:10.1371/journal.pone.0361667)
Supplement: Supplementary file 7 [file pone.0361667.s008.docx]

**Reviewer #1:** 
ABSTRACT
1. The abstract suggests that multiple factors are manipulated (high vs. low calorie cues, dynamic vs. static presentation, and food vs. object stimuli), implying a 2×2×2 design. However, the focus appears to be primarily on dynamic versus static food cues, without addressing the role of calorie content. This discrepancy should be clarified.

We appreciate the reviewer’s comment regarding the unclear study design. We have revised the abstract to clarify that calorie content (high vs. low) was not a fully crossed factor but rather separated across two runs, and that the primary analysis was stimulus format (dynamic vs. static) in lines 38, “Two fMRI runs were conducted: with high-calorie food and object cues presented in Run 1 and low-calorie food and object cues presented in Run 2. Stimulus format (dynamic vs. static) was manipulated within each run, and analyses of format effects were conducted separately for the high- and low-calorie conditions.” We hope that this revision clarifies the structure of the design and the primary focus of the study.

2. In the abstract it should be clear that also non-food cues are included.

We have revised the abstract to explicitly introduce non-food object stimuli early in the description of the paradigm, and have placed references to “object” with “non-food object” throughout, so that their role as a contrast condition is clearer.

INTRODUCTION
3. The introduction would benefit from a clearer explanation of the broader relevance of food cue reactivity, particularly in relation to conditions such as obesity and binge eating disorders.

We have expanded the introduction to more explicitly connect heightened neural responses to food cues with conditions such as obesity or binge eating disorder, recognizing that dysregulated cue reactivity has been implicated in the maintenance of obesogenic eating behaviors and impaired inhibitory control, in lines 68, “Neural responses to food cues vary across individuals and have been linked to overeating and weight-related outcomes. Specifically, greater activation in reward-related regions and decreased inhibitory control have been observed in individuals with obesity and binge eating disorder, potentially contributing to excess caloric intake and dysregulated dietary regulation”. We believe this addition strengthens the motivation for the study and highlights the clinical relevance.

4. The use of dynamic cues is presented as novel. However, dynamic stimuli (e.g., videos) are already widely used in the addiction literature. A more comprehensive discussion of this literature is needed, along with a clearer explanation of how the current study adds to existing work. See for example the following reviews: Starcke et al., 2018 in the context of behavioral addictions, Brobbin et al., 2025 in the context of cocaine use disorder, Jasinska et al., 2014 in the context of substance use disorders in general and Betts et al., 2021 in the context of nicotine use disorder.

Betts, J. M., Dowd, A. N., Forney, M., Hetelekides, E., & Tiffany, S. T. (2021). A Meta-Analysis of Cue Reactivity in Tobacco Cigarette Smokers. Nicotine and Tobacco Research, 23(2), 249–258. [https://doi.org/10.1093/ntr/ntaa147](https://urldefense.com/v3/__https:/doi.org/10.1093/ntr/ntaa147__;!!K-Hz7m0Vt54!lRZ-jQjPhU4rGFaUCiK8iSHirBQU96WrS3-SV386LXlAcUMH2_j_5v5WBujovK_bFbpOHz801lPmPg$)
Brobbin, E., Lowry, N., Cella, M., Copello, A., Coulton, S., Di Pietro, J., Drummond, C., Glautier, S., Kiyak, C., Phillips, T., Stahl, D., Starr, S., Valmaggia, L., Williams, C., & Deluca, P. (2025). Cocaine Cues Used in Experimental Research: A Systematic Review. Brain Sciences, 15(6). [https://doi.org/10.3390/brainsci15060626](https://urldefense.com/v3/__https:/doi.org/10.3390/brainsci15060626__;!!K-Hz7m0Vt54!lRZ-jQjPhU4rGFaUCiK8iSHirBQU96WrS3-SV386LXlAcUMH2_j_5v5WBujovK_bFbpOHz_ZDpczfA$)
Jasinska, A. J., Stein, E. A., Kaiser, J., Naumer, M. J., & Yalachkov, Y. (2014). Factors modulating neural reactivity to drug cues in addiction: A survey of human neuroimaging studies. Neuroscience & Biobehavioral Reviews, 38, 1–16. [https://doi.org/10.1016/j.neubiorev.2013.10.013](https://urldefense.com/v3/__https:/doi.org/10.1016/j.neubiorev.2013.10.013__;!!K-Hz7m0Vt54!lRZ-jQjPhU4rGFaUCiK8iSHirBQU96WrS3-SV386LXlAcUMH2_j_5v5WBujovK_bFbpOHz_qPpZdfg$)
Starcke, K., Antons, S., Trotzke, P., & Brand, M. (2018). Cue-reactivity in behavioral addictions: A meta-analysis and methodological considerations. Journal of Behavioral Addictions, 7(2), 227–238. [https://doi.org/10.1556/2006.7.2018.39](https://urldefense.com/v3/__https:/doi.org/10.1556/2006.7.2018.39__;!!K-Hz7m0Vt54!lRZ-jQjPhU4rGFaUCiK8iSHirBQU96WrS3-SV386LXlAcUMH2_j_5v5WBujovK_bFbpOHz-4ZlJhFQ$)

We thank the reviewer for raising this important point and providing these helpful references. We agree that the use of dynamic stimuli is well established in the addiction literature. We have revised the introduction to discuss the use of dynamic cues in neuroimaging studies of substance and behavioral addictions by adding an additional paragraph starting in lines 80, “The implementation of dynamic cues in research studies is well established in the broader use reactivity literature regarding addiction. In the context of substance use disorders and behavioral addictions, dynamic cues, such as videos depicting drug use and tobacco use, have been shown to elicit stronger subjective craving and greater neural reactivity than static images, engaging regions involved in reward, attention, and motor preparation. However, neuroimaging studies of food cue reactivity have largely relied on static image paradigms. Evaluating dynamic stimuli for visual food cues may better align with the contemporary food environment, which is increasingly dominated by video-based media, and may improve the capacity of experimental paradigms to assess food cue reactivity. In addition, there remains a need to establish and evaluate novel paradigms incorporating dynamic food cues, and to assess whether neural responses to dynamic food cues align with those demonstrated in the literature using static food cues.”

5. Finally, the authors should explicitly state their hypotheses regarding expected differences between static and dynamic cues.

We agree that the introduction did not explicitly state our directional hypothesis, and we have revised it accordingly by adding, in line 106, “We hypothesized that dynamic high-calorie food cues would elicit greater activation in *a priori* reward- and appetite-related ROIs than static high-calorie food cues, using non-food object stimuli as a comparison condition to isolate food-specific responses.”

METHODS
The fMRI task is innovative and promising, but several methodological concerns need to be addressed.

6. First, the sample size (n = 20; n = 17 after exclusions) is considerably smaller than typically recommended. While this may be acceptable for a pilot study, a clear justification for this sample size is required.

Thank you for this comment. We added that the primary purpose of this pilot study was not to provide definitive hypothesis testing but to evaluate feasibility and to obtain preliminary estimates of effect sizes for dynamic versus static food cue contrasts, given that this was our first implementation of this novel paradigm. We have revised the “Study Population and Data Collection” section in the methods by adding the following statement in line 128, “This pilot was designed to evaluate feasibility and to obtain preliminary estimates of effect sizes for dynamic versus static food cue contrasts, which informed the sample size.”

7. Second, three participants were excluded for “falling asleep.” This criterion appears subjective. The authors should clarify how sleep was monitored and what criteria were used for exclusion.

We appreciate the reviewer’s comment regarding the exclusion criterion for inattention. We have added the additional information to clarify how this was assessed (line 123), “Participants were continuously monitored via the camera inside the scanner during scanning. Participants who exhibited sustained eye closure and lack of responsiveness to verbal prompts were classified as inattentive (e.g., falling asleep). Participants whose eyes remained closed for more than three consecutive blocks despite verbal prompts to remain awake were excluded from further analysis.”

8. Third, the task design is inconsistently described. At some points, it appears to include high- and low-calorie food and object cues; elsewhere, only three cue types (high-calorie food, low-calorie food, objects) are described. The design should be consistently and clearly presented as a 3 (cue type: high-calorie, low-calorie, object) × 2 (format: static, dynamic) factorial design
The cues used in the fmri task are either from validated datasets, or have been validated prior to implementing them in the fmri paradigm, which is a strong aspect of the study. A major limitation however is the lack of full randomization. Although static and dynamic blocks were randomized, the task always began with object cues followed by food cues, and high-calorie cues always preceded low-calorie cues. This introduces substantial order effects. The rationale for this design choice is unclear and should be justified, and its limitations should be explicitly discussed.

Thank you for your comment regarding the need for a clearer description of the experimental design. We have revised the Methods and Figure 1 in lines 184, “The task was structured as two parallel 2 x 2 within-run designs, with cue type (food vs. object) and stimulus format (dynamic vs. static) manipulated within each run. High-calorie and low-calorie conditions were presented in separate runs and analyzed independently, rather than as a fully crossed factor.” To better acknowledge this limitation in the design choice, we added that this design limits full counterbalancing and may introduce order effects. We have explicitly noted this as a limitation of the study in the discussion in lines 458, “While this design was implemented to prioritize comparisons of dynamic versus static stimulus format within each run, it may introduce potential order effects, particularly related to the fixed sequencing of dynamic and static blocks and the presentation of high-calorie stimuli in Run 1 and low-calorie stimuli in Run 2, as high-calorie food cues as the primary condition of interest and low-calorie food cues as a secondary condition. Although fixation blocks were included between stimulus blocks to minimize carryover effects, residual influences of prior stimulus exposure may still be present. Future studies should incorporate full randomization of cue type and presentation format to isolate these effects.”

9. Finally, the statistical analysis section lacks sufficient detail. The authors should clearly specify the statistical models used (e.g., linear mixed models), as well as the software and analysis pipeline.

We appreciate the reviewer’s comment regarding the level of detail in the statistical analysis section. We have revised the “Statistical Modeling and Analysis” to clearly specify the statistical models and software used in line 250, “First-level models were estimated using a GLM in FEAT within FSL (version 6.0)” and in line 279, “using FSL’s fslmaths and fslstats. Paired t-tests were conducted in R (version 4.3.1; R Core Team, 2023 to compare mean parameter estimates between dynamic and static food-related contrast (relative to object cues), examined separately for each *a priori* ROI and each calorie cue condition.”

RESULTS
10. In the results, please clarify whether sex-assigned at birth, gender-identity or both were assessed. When assessing sex-assigned at birth, the terminology female/male is correct, but when gender-identity is assessed, the terminology men, women (in addition to non-binary and other) should be used.

We appreciate your insight regarding the clarity of sex and gender terminology. We confirm that participants self-reported their biological sex, therefore, the terminology of female/male is appropriate. We have revised “Study Population and Data Collection” in the methods by adding “Participants self-reported their biological sex” in line 122.

11. The statement that there were no differences in appeal ratings is noteworthy, as I assume that high-calorie foods are typically rated as more appealing. Reporting these data in detail would help interpret the neural findings. Similarly, it would be useful to report any differences in task accuracy across cue types and formats.

Thank you for your insight. We have removed “data not shown” and added a supplementary table reporting mean appeal ratings by presentation format, examined separately within each calorie condition in Supplementary Table 1, with results now described in the result section (line 307), “Among participants included in the analysis (N=17), appeal ratings of dynamic high-calorie, dynamic low-calorie, static high-calorie, and static low-calorie food cues were high overall, ranging from 5.90-6.75 (out of 10) (S2 Table). Appeal ratings were examined separately within each calorie condition and did not differ significantly by stimulus format (static vs. dynamic) or by types of food cues (high vs. low-calorie) for the stimuli presented during the MRI session.”

12. Abbreviations (e.g., HW, OW) should be defined upon first use.

Thank you for your comment. We have defined healthy weight (HW) and overweight (OW) in “Study Population and Data Collection” in the methods in line 122.

13. The current analysis approach appears to rely on difference scores analyzed with t-tests. I recommend instead using linear mixed models to test the main effects of cue type and format, as well as their interaction, while accounting for within-subject dependencies. These methods should be clearly described in the Methods section.

We appreciate the reviewer’s suggestion to use linear mixed models to account for within-subject dependencies. We considered this approach, however, given our within-subject design with two levels of format (dynamic vs. static), a linear mixed model with a random intercept for participant is mathematically equivalent to a paired t-test in this case. To confirm this, we fitted linear mixed models for each ROI, which produced singular fits with random intercept variance estimated at zero, yielding identical t-statistics and p-values to the paired t-tests. This equivalence is expected in balanced two-condition within-subject designs. We have revised the methods section to make the within-subject nature of the analysis more explicit and to clarify that paired t-tests were used to compare dynamic versus static food-related activation within each calorie condition separately (in lines 279, “Paired t-tests were conducted in R (version 4.3.1; R Core Team, 2023 to compare mean parameter estimates between dynamic and static food-related contrast (relative to object cues), examined separately for each *a priori* ROI and each calorie cue condition“. We agree that linear mixed models would be the preferred approach in future studies with more complex designs.

Regarding the suggestion to model cue type (food vs. object) and format (dynamic vs. static) as a 2x2 interaction, we chose the paired t-test for two reasons. First, our primary research question focused on the effect of stimulus format (dynamic vs. static) rather than calorie content (high-cal or low-cal vs. object), and our analyses were designed to address this specific contrast. Second, we note that our current approach is mathematically equivalent to testing this interaction term. Specifically, by first computing the food>object contrast within each format condition, then comparing these contrast estimates between dynamic and static formats, we are directly estimating the format x cue type interaction. Please let us know if you have any further suggestions regarding the statistical approach.

14. For the whole-brain analyses, it appears that interaction contrasts are tested, but main effects are not. The analytical strategy should be clarified and expanded. Specifically, I recommend conducting the following simple effects analyses: High cal>object for dynamic cues only; high cal>object for static cues only; dynamic > static for high cal cues only; dynamic > static for object cues only, as well as testing effects in both directions.

We appreciate the reviewer’s suggestion to expand the whole-brain analyses to include additional contrasts as well as in the negative direction. The following contrasts were examined for both high- and low-calorie conditions:

1. High-calorie food > object for dynamic cues only
2. High-calorie food > object for static cues only
3. Dynamic > static for high-calorie food cues only
4. Dynamic > static for object cues only

Contrasts 1, 2, 3 were already included in Table 2. We have now added contrast 4 to the whole-brain analyses and reported significant clusters in both directions for all contrasts. We have added in the results in line 351, “For object stimuli, dynamic cues elicited greater activation than static cues in bilateral lateral occipital and superior parietal regions (all FDR-corrected *p*’s < 0.011), while static object cues showed greater activation than dynamic cues in occipital, temporal, and motor regions, including the right juxtapositional lobule cortex and right planum polare (all FDR-corrected *p*’s < 0.04). No significant clusters were observed in the static versus dynamic high-calorie food contrast”. Please let us know if you have further suggestions or concerns.

15. The claim that the paradigm was “feasible and well tolerated” is not supported by data. The authors should provide quantitative evidence (e.g., completion rates, engagement accuracy, post-scan feedback). Otherwise, this statement should be removed.

We appreciate the reviewer’s comment and agree that the original statement required clarity. We have revised the result to provide quantitative indicators of feasibility and tolerability. Specifically, we clarified that all 20 enrolled participants completed the full study visit and MRI protocol, while also transparently reporting that three participants were excluded from analysis due to inattention. To support feasibility and engagement, we have added the following in lines 305: “Participants (N=17) correctly identified previously seen images and videos with a mean accuracy on the post-MRI memory task of 78% (SD=13%), reflecting adequate attention and engagement during the scan” and in line 319, “Post scan feedback was generally positive. No participants reported discomfort, nausea, or distress related to the paradigm, indicating good tolerability. The most commonly reported concern was sleepiness during fixation blocks, consistent with previous concerns regarding the passive viewing nature of the task in the confined MRI environment. No runs were terminated early.“

DISCUSSION
16. The discussion currently emphasizes feasibility and tolerability, but supporting evidence is not presented in the Results. The authors should either provide such evidence or refocus the discussion on the main experimental findings.

Thank you for your comment. Similar to comment #15, we have revised the first paragraph of the discussion to provide more adequate evidence of the feasibility and tolerability of the study paradigm to support the feasibility and engagement of this pilot study.

17. The explanations for null findings are somewhat fragmented. The role of sample size should be discussed more rigorously (e.g., observed effect sizes, required sample sizes based on prior literature). Other explanations, such as individual relevance or learning history, should be more clearly elaborated and integrated.

We thank the reviewer for this helpful suggestion and agree that the explanation of the null findings required clarity and rigorous treatment of statistical power. We have revised the Discussion to consolidate the interpretation of the null results, integrating the observed effect sizes and post hoc power considerations in lines 391, “dynamic versus static food cues did not elicit statistically significant differences in activation within *a priori* reward-related ROIs when contrasted with object responses for either high- or low-calorie food conditions. However, these findings should be interpreted with caution given the limited statistical power of this pilot study. Observed effect sizes were negligible to small across most ROIs, suggesting that any true effects of stimulus format are likely modest. Power analyses based on these observed effect sizes indicate that substantially larger sample (e.g., 40 participants or more) would be required to detect effects of this magnitude observed in the dorsal striatum for high-calorie foods with 80% power at 𝛼=0.05. Together, these findings suggest that the present study was underpowered to detect small-to-moderate effects, and therefore should not be interpreted as evidence that stimulus format does not influence reward-related neural responses. Rather, these results provide preliminary estimates of effect size to inform the design and power calculations of future studies.’ We have also expanded the discussion of alternative explanations for the null findings, including the role of individual-level factors such as personal relevance to food cues presented, hunger state, and dietary restraint, and have integrated these more clearly into the broader cue reactivity literature in line 408, “These studies suggest that the salience or personal relevance of the cue depends less on its presentation format and more on factors such as current hunger state, dietary restraint, or prior exposure to specific foods. These findings also suggest that incorporating additional sensory modalities, such as olfactory cues, could capture the multisensory nature of real-world food cue exposure and its effect on reward-related neural processing.”

18. The implications of the findings should also be more thoroughly explored. For example, if dynamic and static cues do not differ meaningfully, what does this imply for future cue-reactivity paradigms? Should alternative modalities (e.g., olfactory cues) be considered?

Similar to our response in #17, we have expanded the discussion of alternative explanations, including individual-level moderators. Regarding the suggestion of alternative sensory modalities, we agree that multisensory cues may engage reward-related regions, and have noted this in the discussion, “These findings also suggest that incorporating additional sensory modalities, such as olfactory cues, could capture the multisensory nature of real-world food cue exposure and its effect on reward-related neural processing” in line 410.

19. The discussion of limitations should be expanded. The restriction to non-obese participants is noted, but the relevance of this paradigm for populations such as individuals with obesity or binge eating disorder should be addressed in more detail.

Thank you for your comment. We agree that the limitation regarding the exclusion of individuals with obesity and eating disorders warranted more thorough discussion. To address the clinical relevance of the paradigm for these population, we have expanded this section in the limitations in line 442, “As individuals with obesity or binge eating disorder may show heightened reward responsivity, altered inhibitory control, and greater cue-induced craving in response to food cues, the present paradigm may capture only a subset of the variability in the neural reactivity that is most clinically relevant for vulnerable populations at elevated risk for overeating. Future studies should assess individuals with obesity and/or eating disorders to examine whether dynamic cue format exacerbates food cue reactivity and whether such responses are associated with clinically meaningful behavioral outcomes.”

20. Finally, the claim that the paradigm is “more naturalistic” is not sufficiently supported. While dynamic cues may increase activation in visual regions, no evidence is provided that they enhance ecologically valid cue reactivity. This conclusion should be more carefully qualified.

We agree that the claim of the paradigm being “more naturalistic” should be more carefully qualified. We have replaced the phrase “more closely reflects real-world food-cue exposure” to “incorporates stimulus formats more representative of contemporary food environment” in line 468 and revised the conclusion to clarify that although dynamic cues elicited stronger neural engagement, the study does not establish enhanced ecological validity in line 473, “However, the extent to which this broader neural engagement translates into greater ecological validity or stronger cue-induced appetitive responses remains to be established.”

**Reviewer #2:** 
Major Comments
1. The analytic strategy would benefit from clearer separation between hypothesis-driven and exploratory approaches. Currently, ROIs are defined using a combination of anatomical masks and functional activation derived from the same dataset. This creates some ambiguity in the analytic framework. For a hypothesis-driven ROI analysis, it would be preferable to define ROIs independently (e.g., purely anatomical masks or regions defined from prior literature), extract the betas and perform the analyses. An alternative approach would be to combine all a priori masks into one mask (let's call it a reward mask) and then look at the effects within that reward mask and extract from the significant clusters within that mask. Also, it would be helpful to show whole brain results rather than just depict them in a table.

We appreciate the reviewer’s thoughtful comment regarding the analytic strategy and the potential circularity introduced by defining functional ROIs from the same dataset. We agree that this warrants clarification and have revised the methods section to more explicitly describe the ROI definition approach (lines 268).

Briefly, the functional ROI definition was based on the overall food, representing all high and low-calorie food>object contrast collapsed across both dynamic and static formats, rather than on the specific dynamic versus static contrast that constitutes the primary outcome of interest. This approach was intentional for two reasons. First, no prior neuroimaging study has examined neural responses to dynamic food cues specifically to our knowledge, meaning that no independently derived functional masks exist for this stimulus type. Second, given that dynamic and static food cues may engage different brain regions, relying solely on anatomical masks or masks derived from previously administered studies utilizing static food cue paradigms could exclude portions of these regions that are responsive to dynamic stimuli but were not previously identified in static paradigms. Using the overall food>object contrast to define ROIs therefore served as an unbiased and data-driven approach to ensure adequate coverage of reward- and appetite-related regions across both presentation formats.

To address the reviewer’s suggested alternative approach, we combined all *a priori* anatomical masks into a single reward mask and examined voxel-wise activation within that mask. This analysis did not yield any additional significant clusters beyond those already reported.

2. High-calorie stimuli were always presented in Run 1 and low-calorie stimuli in Run 2. This fixed order introduces potential confounds (e.g., fatigue, habituation, satiety changes), making it difficult to interpret differences between high- and low-calorie conditions. This limitation should be more clearly acknowledged and discussed.

Thank you for your comment. We agree that the fixed ordering of high-calorie stimuli in Run 1 and low-calorie in Run 2 introduces potential confounds, therefore acknowledge this in the methods, “Block order was partially constrained rather than fully randomized” in line 196, and as an explicit limitation in the limitations paragraph of the discussion in lines 458, “While this design was implemented to prioritize comparisons of dynamic versus static stimulus format within each run, it may introduce potential order effects, particularly related to the fixed sequencing of dynamic and static blocks and the presentation of high-calorie stimuli in Run 1 and low-calorie stimuli in Run 2, as the high-calorie food cues represent the primary condition of interest and low-calorie food cues as a secondary condition. Although fixation blocks were included between stimulus blocks to minimize carryover effects, residual influences of prior stimulus exposure may still be present. Future studies should incorporate full randomization of cue type and presentation format to isolate these effects.”

4.The use of a fixed 4-hour fasting period helps reduce variability in appetitive state; however, this approach may not fully standardize hunger across participants, given individual differences in metabolism and meal composition. It would strengthen the study to report (or control for) additional measures such as time since last meal, meal characteristics, or subjective hunger ratings. Without these measures, it is difficult to determine whether variability in appetitive state may have influenced the neural responses observed. This limits the ability to interpret the neural findings in terms of meaningful differences in appetite or motivation. Please expand the discussion of this limitation.

We appreciate the reviewer’s comment regarding the potential influence of appetitive state variability on neural responses to food cues. We agree that a fixed fasting period alone may not fully standardize hunger across participants. We have expanded the limitations section to address this more explicitly. In addition, we added that pre-scan hunger and fullness ratings did not differ between weight groups across any measures in Supplementary Table 3 and in the results section, in line 314: “Pre-scan hunger ratings were moderately high (mean=56.6, SD=23.6 on a 1-100 scale), while pre-scan fullness ratings were relatively low (mean=21.1, SD=23.9), indicating variability in appetitive state at the start of the scan. Pre-scan hunger and fullness ratings did not differ significantly between individuals with healthy weight and overweight (all *p*’s > 0.05; S3 Table), suggesting that the appetitive state was comparable between weight groups at the time of scan.”

5. The manuscript currently suggests that dynamic cues do not “robustly amplify” reward-related activation. Given the limited power, this conclusion should be softened. The data are inconclusive rather than evidence of no effect.

We appreciate the reviewer’s comment and agree that the interpretation of the null findings should be more cautious, given the limited statistical power. We have revised the discussion to soften the conclusions and clearly convey that the findings are inconclusive rather than evidence of no effect. Specifically, we now state that the results should be interpreted with caution and should not be taken as evidence that stimulus format does not influence reward-related neural responses in lines 391, “dynamic versus static food cues did not elicit statistically significant differences in activation within *a priori* reward-related ROIs when contrasted with object responses for either high- or low-calorie food conditions. However, these findings should be interpreted with caution, given the limited statistical power of this pilot study. Observed effect sizes were negligible to small across most ROIs, suggesting that any true effects of stimulus format are likely modest. Power analyses based on these observed effect sizes indicate that substantially larger sample (e.g., 40 participants or more) would be required to detect effects of this magnitude observed in the dorsal striatum for high-calorie foods with 80% power at 𝛼=0.05. Together, these findings suggest that the present study was underpowered to detect small-to-moderate effects, and therefore should not be interpreted as evidence that stimulus format does not influence reward-related neural responses. Rather, these results provide preliminary estimates of effect size to inform the design and power calculations of future studies”. We have also expanded the discussion of alternative explanations, including the role of individual-level factors, “Beyond statistical power, other factors may contribute to the absence of robust format effects in reward-related regions” in line 403 and “These studies suggest that the salience or personal relevance of the cue depend less on its presentation format or modalities and more on factors such as current hunger state, dietary restraint, or prior exposure to specific foods” in line 408.

6. Given that the dynamic stimulus set is a central contribution of this work, I encourage the authors to consider making the video stimuli publicly available, for example as supplementary materials or via a Github repository. This would substantially enhance reproducibility and facilitate reuse of the paradigm by other researchers.

We appreciate the reviewer’s suggestion. To facilitate replication and reuse of the paradigm, 1) we have added representative example videos for each stimulus category of high-calorie food, low-calorie food, and object as S1 Video, to illustrate the nature and quality of the dynamic stimuli used; 2) we provide a detailed description of the stimulus selection and validation procedure in the methods section, which would allow other researchers to assemble a comparable stimulus set using commercially available stock videos; and 3) we report the specific rating criteria and thresholds used to classify stimuli as high- or low-calorie.

Minor Comments
Please report behavioral results (e.g., appeal ratings) more fully rather than “data not shown.”

We have added an appeal ratings table in Supplementary Table 1 to show the results.
Consider reporting effect sizes (e.g., Cohen’s d) alongside p-values.

Thank you for your suggestion. We have added Cohen’s d in our main tables, Table 1 and Supplementary Table 4, and added in the results: “Observed effect sizes for the dynamic versus static food-related contrast were negligible to small for both high-calorie (d = 0.11 to 0.46) and low-calorie (d = -0.09 to 0.31) conditions, with the dorsal striatum showing the largest effects in both calorie conditions (Table 1 and S4 Table)” in line 330.

**Journal Requirements**

When submitting your revision, we need you to address these additional requirements.

1. Please ensure that your manuscript meets PLOS ONE's style requirements, including those for file naming. The PLOS ONE style templates can be found at

[https://journals.plos.org/plosone/s/file?id=wjVg/PLOSOne_formatting_sample_main_body.pdf](https://urldefense.com/v3/__https:/journals.plos.org/plosone/s/file?id=wjVg*PLOSOne_formatting_sample_main_body.pdf__;Lw!!K-Hz7m0Vt54!lRZ-jQjPhU4rGFaUCiK8iSHirBQU96WrS3-SV386LXlAcUMH2_j_5v5WBujovK_bFbpOHz_edTbiwQ$)

[https://journals.plos.org/plosone/s/file?id=ba62/PLOSOne_formatting_sample_title_authors_affiliations.pdf](https://urldefense.com/v3/__https:/track.editorialmanager.com/CL0/https:*2F*2Fjournals.plos.org*2Fplosone*2Fs*2Ffile*3Fid=ba62*2FPLOSOne_formatting_sample_title_authors_affiliations.pdf/1/010f019d67fb2c64-6aaa85cd-77a3-47a9-bb0c-a6770106f347-000000/6GYWytdPOtRDd0F1EHrIO7G_h1xYU_GUwOWqlFC7K0w=257__;JSUlJSUlJQ!!K-Hz7m0Vt54!lRZ-jQjPhU4rGFaUCiK8iSHirBQU96WrS3-SV386LXlAcUMH2_j_5v5WBujovK_bFbpOHz-kxIjFIQ$)

Thank you for the guidance regarding PLOS ONE’s style requirements. We have revised the manuscript and updated the files to follow the style templates. If there are any specific elements that still need adjustment, please let us know and we will correct them promptly.

2. We note that the grant information you provided in the ‘Funding Information’ and ‘Financial Disclosure’ sections do not match.

When you resubmit, please ensure that you provide the correct grant numbers for the awards you received for your study in the ‘Funding Information’ section.

Thank you for the note regarding the funding information. We reviewed the “Grant Support” section in the manuscript and the “Funding Information” fields in the submission system, and the grant numbers appear consistent across both locations. To ensure full alignment in the resubmission, could you indicate which specific grant number of entry appears mismatched on your end? We will be happy to correct it immediately.

3. Thank you for stating the following financial disclosure:
Support for this work was provided by grants from the National Institutes of Health K24HL144917 (Ellen Schur), R01DK117623 (Ellen Schur), R01DK089036 (Ellen Schur), the University of Washington Nutrition and Obesity Research Center P30DK035816, and the American Heart Association grant 929228 (Leticia Sewaybricker) and 26POST1550949 (Dabin Yeum).

Please state what role the funders took in the study. If the funders had no role, please state: "The funders had no role in study design, data collection and analysis, decision to publish, or preparation of the manuscript."
If this statement is not correct you must amend it as needed.
Please include this amended Role of Funder statement in your cover letter; we will change the online submission form on your behalf.

We have added “The funders had no role in study design, data collection and analysis, decision to publish, or preparation of the manuscript” in “Grant support” as part of the manuscript and in the cover letter.

4. Thank you for stating the following in the Competing Interests/Financial Disclosure section:
Dr. Ellen Schur discloses past consulting work for Amgen.

We note that one or more of the authors are employed by a commercial company: Amgen

a. Please provide an amended Funding Statement declaring this commercial affiliation, as well as a statement regarding the Role of Funders in your study. If the funding organization did not play a role in the study design, data collection and analysis, decision to publish, or preparation of the manuscript and only provided financial support in the form of authors' salaries and/or research materials, please review your statements relating to the author contributions, and ensure you have specifically and accurately indicated the role(s) that these authors had in your study. You can update author roles in the Author Contributions section of the online submission form.

Please also include the following statement within your amended Funding Statement.
“The funder provided support in the form of salaries for authors [insert relevant initials], but did not have any additional role in the study design, data collection and analysis, decision to publish, or preparation of the manuscript. The specific roles of these authors are articulated in the ‘author contributions’ section.”
If your commercial affiliation did play a role in your study, please state and explain this role within your updated Funding Statement.

b. Please also provide an updated Competing Interests Statement declaring this commercial affiliation along with any other relevant declarations relating to employment, consultancy, patents, products in development, or marketed products, etc.

Within your Competing Interests Statement, please confirm that this commercial affiliation does not alter your adherence to all PLOS ONE policies on sharing data and materials by including the following statement: "This does not alter our adherence to PLOS ONE policies on sharing data and materials.” (as detailed online in our guide for authors [http://journals.plos.org/plosone/s/competing-interests](https://urldefense.com/v3/__http:/journals.plos.org/plosone/s/competing-interests__;!!K-Hz7m0Vt54!lRZ-jQjPhU4rGFaUCiK8iSHirBQU96WrS3-SV386LXlAcUMH2_j_5v5WBujovK_bFbpOHz_ThZ1-ow$)) . If this adherence statement is not accurate and there are restrictions on sharing of data and/or materials, please state these. Please note that we cannot proceed with consideration of your article until this information has been declared.

Please include both an updated Funding Statement and Competing Interests Statement in your cover letter. We will change the online submission form on your behalf.

Dr. Ellen Schur had past consulting work for Amgen that ended in 2023, and she was not employed by or received any salary from Amgen. Amgen had no involvement in this study in any capacity. Please let us know if you need further clarification or if we still need to submit the Funding Statement and Competing Interests Statement.

5. We note that you have indicated that there are restrictions to data sharing for this study. For studies involving human research participant data or other sensitive data, we encourage authors to share de-identified or anonymized data. However, when data cannot be publicly shared for ethical reasons, we allow authors to make their data sets available upon request. For information on unacceptable data access restrictions, please see [http://journals.plos.org/plosone/s/data-availability#loc-unacceptable-data-access-restrictions](https://urldefense.com/v3/__http:/journals.plos.org/plosone/s/data-availability*loc-unacceptable-data-access-restrictions__;Iw!!K-Hz7m0Vt54!lRZ-jQjPhU4rGFaUCiK8iSHirBQU96WrS3-SV386LXlAcUMH2_j_5v5WBujovK_bFbpOHz9Zs0yGBw$).

Before we proceed with your manuscript, please address the following prompts:

a) If there are ethical or legal restrictions on sharing a de-identified data set, please explain them in detail (e.g., data contain potentially identifying or sensitive patient information, data are owned by a third-party organization, etc.) and who has imposed them (e.g., a Research Ethics Committee or Institutional Review Board, etc.). Please also provide contact information for a data access committee, ethics committee, or other institutional body to which data requests may be sent.

b) If there are no restrictions, please upload the minimal anonymized data set necessary to replicate your study findings to a stable, public repository and provide us with the relevant URLs, DOIs, or accession numbers. Please see [http://www.bmj.com/content/340/bmj.c181.long](https://urldefense.com/v3/__http:/www.bmj.com/content/340/bmj.c181.long__;!!K-Hz7m0Vt54!lRZ-jQjPhU4rGFaUCiK8iSHirBQU96WrS3-SV386LXlAcUMH2_j_5v5WBujovK_bFbpOHz8vkdgNtg$) for guidelines on how to de-identify and prepare clinical data for publication. For a list of recommended repositories, please see [https://journals.plos.org/plosone/s/recommended-repositories](https://urldefense.com/v3/__https:/journals.plos.org/plosone/s/recommended-repositories__;!!K-Hz7m0Vt54!lRZ-jQjPhU4rGFaUCiK8iSHirBQU96WrS3-SV386LXlAcUMH2_j_5v5WBujovK_bFbpOHz91BZmPRw$). You also have the option of uploading the data as Supporting Information files, but we would recommend depositing data directly to a data repository if possible.

Please update your Data Availability statement in the submission form accordingly.

We thank the editor for the guidance regarding data sharing. Since our initial submission, we have amended our IRB protocol at the University of Washington to permit sharing of de-identified aggregated data. A deidentified, aggregated dataset has been deposited in Zenodo and will be made publicly available upon publication with the doi: 10.5281/zenodo.19703232 and with the full citation “Yeum, D., Melhorn, S., Lao, C., Anderson, C., Rane, S., Schur, E., & Sewaybricker, L. (2026). Dataset: Neural food cue reactivity fMRI pilot study — ROI parameter estimates and covariates [Data set]. Zenodo. <https://doi.org/10.5281/zenodo.19703232>”.

Regarding the raw fMRI data, these cannot be made publicly available due to the potential for re-identification inherent in human neuroimaging data, including structural MRI scans. Restrictions on sharing raw imaging data were imposed by our IRB at the University of Washington. Researchers interested in accessing the raw imaging data may submit a request to the principal investigator below, and data sharing will be considered on a case-by-case basis in accordance with our IRB protocol:

Leticia E. Sewaybricker / Department of General Internal Medicine / [leticker@uw.edu](mailto:leticker@uw.edu)

We updated the Data Availability statement in the submission page to reflect these arrangements accordingly.

6. Please amend the manuscript submission data (via Edit Submission) to include author Cindy Lao

Thank you for the note regarding the author list. I currently see Cindy Lao included in the “Current Author List” on the manuscript submissions page. Please let us know if the issue persists. We will make the correction immediately.

7. Please amend your authorship list in your manuscript file to include author Cindy Lao

Similar to the issue above, I see Cindy Lao included in the author byline in the manuscript file I uploaded as well as the PDF receipt. Please let us know if you still don’t see Cindy Lao in the file. We will make the correction immediately.

8. Please amend your list of authors on the manuscript to ensure that each author is linked to an affiliation. Authors’ affiliations should reflect the institution where the work was done (if authors moved subsequently, you can also list the new affiliation stating “current affiliation:….” as necessary).

We have added and confirmed each author’s affiliation in the submission system, and in the manuscript file each author is linked to their affiliation using superscript numbers. Please let us know if the author or affiliation appears mismatched on your end.

9. We note that you have included the phrase “data not shown” in your manuscript. Unfortunately, this does not meet our data sharing requirements. PLOS does not permit references to inaccessible data. We require that authors provide all relevant data within the paper, Supporting Information files, or in an acceptable, public repository. Please add a citation to support this phrase or upload the data that corresponds with these findings to a stable repository (such as Figshare or Dryad) and provide and URLs, DOIs, or accession numbers that may be used to access these data. Or, if the data are not a core part of the research being presented in your study, we ask that you remove the phrase that refers to these data.

We thank the editor for this note. We have removed the phrase “data not shown” from the manuscript and have added the corresponding data as Supplementary Table 1, which reports appeal ratings by presentation format examined separately within each calorie condition. All relevant data are now accessible within the manuscript or its supporting information files.

10. If the reviewer comments include a recommendation to cite specific previously published works, please review and evaluate these publications to determine whether they are relevant and should be cited. There is no requirement

We have reviewed all publications recommended by the reviewers and have incorporated them accordingly in our manuscript to expand the scope of the introduction and discussion. References that were not directly relevant to the work were not included.
