# Supplementary material for: Neural responses to dynamic versus static food cues: A pilot functional MRI study
Source: PLoS One. 2026 Oct 28;21(10):e0361667. doi: 10.1371/journal.pone.0361667 (PMC13716230; doi:10.1371/journal.pone.0361667)
Supplement: Supplementary file 8 [file pone.0361667.s009.docx]

**Due 8/8**

**Additional Editor Comments:**
Comment 1:
Reference 18 (Devoto F.) is incomplete — journal name, volume, pages, and DOI are all missing. Please provide the full citation.

Response: Thank you for catching the incomplete citation. We have revised the citation to include the full citation information.

Comment 2:
References 7 and 20 are identical (Mehta et al., 2012, AJCN), as are References 8 and 21 (Schur et al., 2009, Int J Obes). Please consolidate these duplicate citations and renumber all references accordingly.

Response: Thank you for bringing this error to our attention. This has now been corrected, and the full list of references are reviewed and adjusted.

Comment 3:
The functional ROI definition was based on food vs. object contrasts (p<0.05, uncorrected) to identify active voxels, after which dynamic vs. static format effects were tested within those same ROIs. Please clarify in the Methods or Discussion whether this approach introduces circularity (double-dipping), and if so, how the independence of the primary analysis was maintained.

Response: We appreciate this comment regarding potential circularity in the ROI definition strategy. We would like to clarify that the functional ROI definition was based on the overall food>object contrast for which we collapsed across all food categories (high and low-calorie foods) and included activation by both dynamic and static stimulus formats (line 268); this was done purposefully to identify a minimal level of responsiveness to food and avoid our specific dynamic versus static contrast that constituted the primary outcome of interest. This orthogonality between the ROI-defining contrast and the primary analysis contrast minimizes the risk of circularity, as the ROI selection was not biased toward the specific effect being tested. We have added a clarifying sentence to the discussion to make this explicit, in lines 462-465, “Although functional ROIs were derived from the same dataset, circularity was minimized by collapsing across all food categories and stimulus formats to define the ROI mask, thereby identifying a minimal level of responsiveness to food cues while avoiding the introduction of contrast-specific bias into the primary dynamic-versus-static analysis.”

Comment 4:
Exploratory weight-group comparisons were conducted across multiple ROIs and calorie conditions without correction for multiple comparisons. Although labeled "exploratory," the reported p-values (e.g., p=.020, p=.007) risk being overinterpreted. Please explicitly state in the Results and/or Discussion that these p-values are uncorrected and should be regarded as hypothesis-generating only.

Response: Thank you for your suggestion. We agree that results from exploratory weight-group comparisons need caution interpretation; therefore, we revised the results section to explicitly state that these p-values are uncorrected in lines 379 and 380, “These exploratory comparisons were not corrected for multiple comparisons”. Also, we have added a clarifying statement to the discussion emphasizing that these comparisons were not corrected for multiple comparisons and were hypothesis-generating only in lines 466-468, “with fewer than 10 participants per group, limiting power to detect true group differences. Although prespecified as exploratory, these findings should be interpreted strictly as hypothesis-generating and were not corrected for multiple comparisons”.

**Reviewer #3**
1. I agree with the overall premise that dynamic vs. static food cues should be investigated and that these responses may differ in individuals who are overweight compared to those who are not. However, if the study was designed to answer the latter question, it is incredibly underpowered, even as a pilot study, with less than 10 participants in each group. While this is presented as exploratory in the last paragraph, I think this should be emphasized more given the extremely limited power to answer the question presented as a gap in the introduction.

Response: We thank the reviewer for this important point that the weight-status comparisons were underpowered, with fewer than 10 participants per group. While these analyses were prespecified as exploratory, we agree that their preliminary and hypothesis-generating nature should be emphasized throughout the manuscript. We have revised the limitations section to more explicitly acknowledge the limited power for weight-status comparisons in the discussion, in lines 466-468, “with fewer than 10 participants per group, limiting power to detect true group differences. Although prespecified as exploratory, these findings should be interpreted strictly as hypothesis-generating and were not corrected for multiple comparisons.”

2. How was pre-scan fasting verified?

Response: We appreciate this question. Participant fasting was reinforced via reminder communication prior to the study visit and verified by verbal self-report (i.e., verbal confirmation of time of last meal to confirm at least a 4-hour fasting interval) upon arrival. We have revised the methods section in lines 119-120 to include this information. We acknowledge that verbal self-report is a limitation and does not establish objective verification of fasting status. In the discussion, we address this limitation in line 487, that “although a standardized 4-hour fasting period was implemented to reduce variability in appetitive state, this approach may not fully equalize hunger across participants given individual differences in metabolism and meal composition”.

3. 2-seconds per stimulus for the dynamic cues is quite short. Were participants able to truly experience the dynamic nature of the cues in that time-frame

Response: We appreciate this important observation. The 2-second presentation duration was a deliberate methodological choice designed to balance experimental control with ecological validity; however, it warrants explicit justification and acknowledgment as a limitation. We have incorporated discussion into the limitation section of Discussion, in lines 501-509, “Lastly, the 2-second presentation duration for dynamic video stimuli was chosen deliberately to match the static image duration, ensuring experimental control and comparability across formats. Furthermore, this brief duration mirrors the short-form media clips and GIFs in the digital food environments. While this brief exposure minimizes potential habituation from rapid serial stimulus transitions during the MRI scan, it may have limited the depth to which the full dynamic nature of certain cues could be integrated. Future studies should systematically evaluate how varying presentation durations for dynamic food cue paradigms impact neural responses.”

4. Effect sizes should be provided for the weight group analyses.

Response: Thank you for this important note. We have updated the manuscript to explicitly include the effect sizes for these analyses. In lines 374 and 379, we now report the test statistic, p-value, and the effect size (Cohen’s d = 1.26 and -1.52) for the weight group comparison. Additionally, the corresponding effect sizes for all ROIs have been added to Table S6.

5. While I agree that findings related to dynamic vs. static images for the ROI analyses should be interpreted with caution, the fact that effect sizes were so small and a large sample would be needed to detect effects indicates that the image state (at least for very short videos) is not a very important factor for studying reward/satiation responses to food cues in the brain.

Response: We appreciate this thoughtful interpretation of the findings. We agree that the small effect sizes observed across most ROIs suggest that stimulus format may have only modest effects on reward-related neural response at the group level. In response to this comment, we revised the Discussion to more clearly acknowledge this interpretation. We now emphasize that the overall similarity of responses to dynamic and static food cues supports the relevance of findings from the existing static-image literature while also highlighting the feasibility and tolerability of using more ecologically valid dynamic stimuli. We additionally note that exploratory subgroup findings suggest the possibility of weight-specific effects that warrant further investigation.

The revised Discussion (lines 399-415) now states, “Observed effect sizes were negligible to small across most ROIs, suggesting that format-related differences in reward-related neural responses to food cues may be modest at on average across all participants. Notably, the broadly similar pattern of activation across dynamic and static food cues suggests that findings from the extensive literature on static food images may remain relevant when interpreting studies that employ more ecologically valid dynamic stimuli. This is encouraging given that one of the primary goals of the present study was to establish the feasibility and tolerability of a dynamic food cue paradigm that more closely reflects food-related media encountered in contemporary environments. Additionally, exploratory analyses identified weight-status differences in neural responses to dynamic versus static food cues in the dorsal striatum for high-calorie foods and in the substantia nigra/ventral tegmental area for low-calorie foods, suggesting that stimulus format may be more relevant in specific subpopulations than in the overall sample. Power analyses based on these observed effect sizes indicate that substantially larger sample (e.g., 40 participants or more) would be required to detect effects of this magnitude. Together, these findings suggest that format-related effects are modest compared to the overall consistently demonstrated robust responsiveness to visual food stimuli and that preliminary effect size estimates support both the use of dynamic stimuli and future studies of potential subgroup-specific differences.

“
