# Supplementary material for: Neural responses to dynamic versus static food cues: A pilot functional MRI study
Source: PLoS One. 2026 Oct 28;21(10):e0361667. doi: 10.1371/journal.pone.0361667 (PMC13716230; doi:10.1371/journal.pone.0361667)
Supplement: Supplementary file 9 [file pone.0361667.s010.docx]

1.Clarify the whole-brain findings in the Abstract: Please distinguish the dynamic-versus-static food-specific contrast, relative to objects, from the food-versus-object contrasts. The former showed significant activation in the right occipital fusiform gyrus for high-calorie foods. Findings in frontal, insular, and cingulate regions should be attributed to their respective contrasts rather than presented collectively as dynamic-versus-static effects.

Response: We appreciate the editor’s request for clarification. We revised the abstract to clearly distinguish whole-brain cluster activation for each contrast. In lines 50-52, we have revised the text to “Whole-brain analyses showed that the dynamic-versus-static high-calorie food contrast (relative to objects) elicited greater activation in the right occipital fusiform gyrus. Frontal, insular, and cingulate activation was observed to the dynamic food-versus-object contrast.”

2. Correct and clarify the effect-size reporting: The high-calorie effect-size range reported at line 333 (d = 0.11–0.46) does not match Table 1, which includes values from −0.11 to 0.46. Please ensure consistency between the text and table, revise the description “negligible to small” to reflect the reported magnitudes, and specify how Cohen’s d was calculated for the paired comparisons.
Response: We thank the editor for catching this inconsistency. The negative sign was added to the range in line 340: “d=-0.11 to 0.46”, consistent with Table 1. We have also added a clarification to the methods specifying the Cohen’s d calculation in lines 289-291, “Effect sizes were calculated as Cohen’s d for paired comparisons, computed as the mean differences between dynamic and static conditions divided by the standard deviation of the differences.” We confirm that the description “negligible to small” accurately reflects the corrected range of d=-0.11 to 0.46, consistent with conventional Cohen’s d benchmarks (negligible <0.2, small=0.2-0.5), and that the omission of the negative sign was the source of the apparent discrepancy.

3. Clarify the sample-size statement: For the statement that “40 participants or more” would be required, please provide the effect size, statistical test, significance level, and target power used in the calculation. Alternatively, remove the numerical estimate and retain a general statement that larger, adequately powered studies are needed.
Response: We appreciate the editor’s request for clarification. We have revised the power analysis statement in the discussion (lines 417-419) to specify the effect size used (d=0.46, observed in the dorsal striatum for high-calorie foods), statistical test (paired t-test), significance level, and target power used in the calculation, “Power analyses based on these observed effect sizes in the dorsal striatum for high-calorie foods (d = 0.46), using a paired t-test at α = 0.05 with 80% power, indicate that approximately 40 participants would be required to detect effects of this magnitude.”
