# Supplementary material for: Neural responses to dynamic versus static food cues: A pilot functional MRI study
Source: PLoS One. 2026 Oct 28;21(10):e0361667. doi: 10.1371/journal.pone.0361667 (PMC13716230; doi:10.1371/journal.pone.0361667)
Supplement: Peer review history

# Peer review: Decision Letter 0

**DOI:** 10.1371/journal.pone.0361667.r001

**Authors:** Yoko Hasegawa

7 Apr 2026

Dear Dr. Yeum,

Thank you for submitting your manuscript to PLOS ONE. After careful consideration, we feel that it has merit but does not fully meet PLOS ONE’s publication criteria as it currently stands. Therefore, we invite you to submit a revised version of the manuscript that addresses the points raised during the review process.

Please submit your revised manuscript by May 22 2026 11:59PM. If you will need more time than this to complete your revisions, please reply to this message or contact the journal office at plosone@plos.org. When you're ready to submit your revision, log on to https://www.editorialmanager.com/pone/ and select the 'Submissions Needing Revision' folder to locate your manuscript file.

- A letter that responds to each point raised by the academic editor and reviewer(s). You should upload this letter as a separate file labeled 'Response to Reviewers'.
- A marked-up copy of your manuscript that highlights changes made to the original version. You should upload this as a separate file labeled 'Revised Manuscript with Track Changes'.
- An unmarked version of your revised paper without tracked changes. You should upload this as a separate file labeled 'Manuscript'.

If you would like to make changes to your financial disclosure, please include your updated statement in your cover letter. Guidelines for resubmitting your figure files are available below the reviewer comments at the end of this letter.

If applicable, we recommend that you deposit your laboratory protocols in protocols.io to enhance the reproducibility of your results. Protocols.io assigns your protocol its own identifier (DOI) so that it can be cited independently in the future. For instructions see: https://journals.plos.org/plosone/s/submission-guidelines#loc-laboratory-protocols. Additionally, PLOS ONE offers an option for publishing peer-reviewed Lab Protocol articles, which describe protocols hosted on protocols.io. Read more information on sharing protocols at https://plos.org/protocols?utm_medium=editorial-email&utm_source=authorletters&utm_campaign=protocols.

As the corresponding author, your ORCID iD is verified in the submission system and will appear in the published article. PLOS supports the use of ORCID, and we encourage all coauthors to register for an ORCID iD and use it as well. Please encourage your coauthors to verify their ORCID iD within the submission system before final acceptance, as unverified ORCID iDs will not appear in the published article. *Only*  the individual author can complete the verification step; PLOS staff *cannot*  verify ORCID iDs on behalf of authors.

We look forward to receiving your revised manuscript.

Kind regards,

Yoko Hasegawa

Academic Editor

PLOS One

Journal Requirements:

When submitting your revision, we need you to address these additional requirements.

1. Please ensure that your manuscript meets PLOS ONE's style requirements, including those for file naming. The PLOS ONE style templates can be found at

https://journals.plos.org/plosone/s/file?id=wjVg/PLOSOne_formatting_sample_main_body.pdf and

https://journals.plos.org/plosone/s/file?id=ba62/PLOSOne_formatting_sample_title_authors_affiliations.pdf

2. We note that the grant information you provided in the ‘Funding Information’ and ‘Financial Disclosure’ sections do not match.

When you resubmit, please ensure that you provide the correct grant numbers for the awards you received for your study in the ‘Funding Information’ section.

3. Thank you for stating the following financial disclosure:

Support for this work was provided by grants from the National Institutes of Health K24HL144917 (Ellen Schur), R01DK117623 (Ellen Schur), R01DK089036 (Ellen Schur), the University of Washington Nutrition and Obesity Research Center P30DK035816, and the American Heart Association grant 929228 (Leticia Sewaybricker) and 26POST1550949 (Dabin Yeum).

Please state what role the funders took in the study. If the funders had no role, please state: "The funders had no role in study design, data collection and analysis, decision to publish, or preparation of the manuscript."

If this statement is not correct you must amend it as needed.

Please include this amended Role of Funder statement in your cover letter; we will change the online submission form on your behalf.

4. Thank you for stating the following in the Competing Interests/Financial Disclosure section:

Dr. Ellen Schur discloses past consulting work for Amgen.

We note that one or more of the authors are employed by a commercial company: Amgen

a. Please provide an amended Funding Statement declaring this commercial affiliation, as well as a statement regarding the Role of Funders in your study. If the funding organization did not play a role in the study design, data collection and analysis, decision to publish, or preparation of the manuscript and only provided financial support in the form of authors' salaries and/or research materials, please review your statements relating to the author contributions, and ensure you have specifically and accurately indicated the role(s) that these authors had in your study. You can update author roles in the Author Contributions section of the online submission form.

Please also include the following statement within your amended Funding Statement.

“The funder provided support in the form of salaries for authors [insert relevant initials], but did not have any additional role in the study design, data collection and analysis, decision to publish, or preparation of the manuscript. The specific roles of these authors are articulated in the ‘author contributions’ section.”

If your commercial affiliation did play a role in your study, please state and explain this role within your updated Funding Statement.

b. Please also provide an updated Competing Interests Statement declaring this commercial affiliation along with any other relevant declarations relating to employment, consultancy, patents, products in development, or marketed products, etc.

Within your Competing Interests Statement, please confirm that this commercial affiliation does not alter your adherence to all PLOS ONE policies on sharing data and materials by including the following statement: "This does not alter our adherence to PLOS ONE policies on sharing data and materials.” (as detailed online in our guide for authors http://journals.plos.org/plosone/s/competing-interests) . If this adherence statement is not accurate and there are restrictions on sharing of data and/or materials, please state these. Please note that we cannot proceed with consideration of your article until this information has been declared.

Please include both an updated Funding Statement and Competing Interests Statement in your cover letter. We will change the online submission form on your behalf.

5. We note that you have indicated that there are restrictions to data sharing for this study. For studies involving human research participant data or other sensitive data, we encourage authors to share de-identified or anonymized data. However, when data cannot be publicly shared for ethical reasons, we allow authors to make their data sets available upon request. For information on unacceptable data access restrictions, please see http://journals.plos.org/plosone/s/data-availability#loc-unacceptable-data-access-restrictions.

Before we proceed with your manuscript, please address the following prompts:

a) If there are ethical or legal restrictions on sharing a de-identified data set, please explain them in detail (e.g., data contain potentially identifying or sensitive patient information, data are owned by a third-party organization, etc.) and who has imposed them (e.g., a Research Ethics Committee or Institutional Review Board, etc.). Please also provide contact information for a data access committee, ethics committee, or other institutional body to which data requests may be sent.

b) If there are no restrictions, please upload the minimal anonymized data set necessary to replicate your study findings to a stable, public repository and provide us with the relevant URLs, DOIs, or accession numbers. Please see http://www.bmj.com/content/340/bmj.c181.long for guidelines on how to de-identify and prepare clinical data for publication. For a list of recommended repositories, please see https://journals.plos.org/plosone/s/recommended-repositories. You also have the option of uploading the data as Supporting Information files, but we would recommend depositing data directly to a data repository if possible.

Please update your Data Availability statement in the submission form accordingly.

6. Please amend the manuscript submission data (via Edit Submission) to include author Cindy Lao

7. Please amend your authorship list in your manuscript file to include author Cinday Lao

8. Please amend your list of authors on the manuscript to ensure that each author is linked to an affiliation. Authors’ affiliations should reflect the institution where the work was done (if authors moved subsequently, you can also list the new affiliation stating “current affiliation:….” as necessary).

9. We note that you have included the phrase “data not shown” in your manuscript. Unfortunately, this does not meet our data sharing requirements. PLOS does not permit references to inaccessible data. We require that authors provide all relevant data within the paper, Supporting Information files, or in an acceptable, public repository. Please add a citation to support this phrase or upload the data that corresponds with these findings to a stable repository (such as Figshare or Dryad) and provide and URLs, DOIs, or accession numbers that may be used to access these data. Or, if the data are not a core part of the research being presented in your study, we ask that you remove the phrase that refers to these data.

10. If the reviewer comments include a recommendation to cite specific previously published works, please review and evaluate these publications to determine whether they are relevant and should be cited. There is no requirement to cite these works unless the editor has indicated otherwise.

Additional Editor Comments:

Thank you for submitting your manuscript entitled “Neural responses to dynamic versus static food cues: A pilot functional MRI study” to PLOS ONE.

Based on the reviewers’ evaluations, we believe your study addresses an interesting and timely research question and includes a valuable methodological contribution through the development of a dynamic food-cue paradigm. However, both reviewers identified several methodological and interpretive issues—particularly concerning ROI definition, stimulus presentation order, and data availability—that need to be addressed before the manuscript can be reconsidered for publication.

We therefore invite you to revise and resubmit your manuscript, responding point-by-point to the reviewers’ comments. Please pay particular attention to clarifying the analytic framework, addressing potential confounds related to the experimental design, and expanding the discussion of study limitations and data availability.

[Note: HTML markup is below. Please do not edit.]

Reviewers' comments:

Reviewer's Responses to Questions

**Comments to the Author**

1. Is the manuscript technically sound, and do the data support the conclusions?

Reviewer #1: Partly

Reviewer #2: Partly

**********

2. Has the statistical analysis been performed appropriately and rigorously?

Reviewer #1: I Don't Know

Reviewer #2: Yes

**********

3. Have the authors made all data underlying the findings in their manuscript fully available?

The PLOS Data policy

Reviewer #1: No

Reviewer #2: No

**********

4. Is the manuscript presented in an intelligible fashion and written in standard English?

Reviewer #1: Yes

Reviewer #2: Yes

**********

Reviewer #1: GENERAL COMMENTS

The authors present an innovative cue-reactivity paradigm with potential applications beyond food research, including addiction studies. The manuscript is generally well written. However, several critical issues including those related to task design and the small sample size substantially limit the strength of the conclusions. In addition, some aspects of the study design and reporting require clarification.

ABSTRACT

The abstract suggests that multiple factors are manipulated (high vs. low calorie cues, dynamic vs. static presentation, and food vs. object stimuli), implying a 2×2×2 design. However, the focus appears to be primarily on dynamic versus static food cues, without addressing the role of calorie content. This discrepancy should be clarified.

In the abstract it should be clear that also non-food cues are included.

INTRODUCTION

The introduction would benefit from a clearer explanation of the broader relevance of food cue reactivity, particularly in relation to conditions such as obesity and binge eating disorders.

The use of dynamic cues is presented as novel. However, dynamic stimuli (e.g., videos) are already widely used in the addiction literature. A more comprehensive discussion of this literature is needed, along with a clearer explanation of how the current study adds to existing work. See for example the following reviews: Starcke et al., 2018 in the context of behavioral addictions, Brobbin et al., 2025 in the context of cocaine use disorder, Jasinska et al., 2014 in the context of substance use disorders in general and Betts et al., 2021 in the context of nicotine use disorder.

Betts, J. M., Dowd, A. N., Forney, M., Hetelekides, E., & Tiffany, S. T. (2021). A Meta-Analysis of Cue Reactivity in Tobacco Cigarette Smokers. Nicotine and Tobacco Research, 23(2), 249–258. https://doi.org/10.1093/ntr/ntaa147

Brobbin, E., Lowry, N., Cella, M., Copello, A., Coulton, S., Di Pietro, J., Drummond, C., Glautier, S., Kiyak, C., Phillips, T., Stahl, D., Starr, S., Valmaggia, L., Williams, C., & Deluca, P. (2025). Cocaine Cues Used in Experimental Research: A Systematic Review. Brain Sciences, 15(6). https://doi.org/10.3390/brainsci15060626

Jasinska, A. J., Stein, E. A., Kaiser, J., Naumer, M. J., & Yalachkov, Y. (2014). Factors modulating neural reactivity to drug cues in addiction: A survey of human neuroimaging studies. Neuroscience & Biobehavioral Reviews, 38, 1–16. https://doi.org/10.1016/j.neubiorev.2013.10.013

Starcke, K., Antons, S., Trotzke, P., & Brand, M. (2018). Cue-reactivity in behavioral addictions: A meta-analysis and methodological considerations. Journal of Behavioral Addictions, 7(2), 227–238. https://doi.org/10.1556/2006.7.2018.39

Finally, the authors should explicitly state their hypotheses regarding expected differences between static and dynamic cues.

METHODS

The fMRI task is innovative and promising, but several methodological concerns need to be addressed.

First, the sample size (n = 20; n = 17 after exclusions) is considerably smaller than typically recommended. While this may be acceptable for a pilot study, a clear justification for this sample size is required.`

Second, three participants were excluded for “falling asleep.” This criterion appears subjective. The authors should clarify how sleep was monitored and what criteria were used for exclusion.

Third, the task design is inconsistently described. At some points, it appears to include high- and low-calorie food and object cues; elsewhere, only three cue types (high-calorie food, low-calorie food, objects) are described. The design should be consistently and clearly presented as a 3 (cue type: high-calorie, low-calorie, object) × 2 (format: static, dynamic) factorial design

The cues used in the fmri task are either from validated datasets, or have been validated prior to implementing them in the fmri paradigm, which is a strong aspect of the study. A major limitation however is the lack of full randomization. Although static and dynamic blocks were randomized, the task always began with object cues followed by food cues, and high-calorie cues always preceded low-calorie cues. This introduces substantial order effects. The rationale for this design choice is unclear and should be justified, and its limitations should be explicitly discussed.

Finally, the statistical analysis section lacks sufficient detail. The authors should clearly specify the statistical models used (e.g., linear mixed models), as well as the software and analysis pipeline.

RESULTS

In the results, please clarify whether sex-assigned at birth, gender-identity or both were assessed. When assessing sex-assigned at birth, the terminology female/male is correct, but when gender-identity is assessed, the terminology men, women (in addition to non-binary and other) should be used.

The statement that there were no differences in appeal ratings is noteworthy, as I assume that high-calorie foods are typically rated as more appealing. Reporting these data in detail would help interpret the neural findings. Similarly, it would be useful to report any differences in task accuracy across cue types and formats.

Abbreviations (e.g., HW, OW) should be defined upon first use.

The current analysis approach appears to rely on difference scores analyzed with t-tests. I recommend instead using linear mixed models to test the main effects of cue type and format, as well as their interaction, while accounting for within-subject dependencies. These methods should be clearly described in the Methods section.

For the whole-brain analyses, it appears that interaction contrasts are tested, but main effects are not. The analytical strategy should be clarified and expanded. Specifically, I recommend conducting the following simple effects analyses: High cal>object for dynamic cues only; high cal?object for static cues only; dynamic > static for high cal cues only; dynamic > static for object cues only, as well as testing effects in both directions.

The claim that the paradigm was “feasible and well tolerated” is not supported by data. The authors should provide quantitative evidence (e.g., completion rates, engagement accuracy, post-scan feedback). Otherwise, this statement should be removed.

DISCUSSION

The discussion currently emphasizes feasibility and tolerability, but supporting evidence is not presented in the Results. The authors should either provide such evidence or refocus the discussion on the main experimental findings.

The explanations for null findings are somewhat fragmented. The role of sample size should be discussed more rigorously (e.g., observed effect sizes, required sample sizes based on prior literature). Other explanations, such as individual relevance or learning history, should be more clearly elaborated and integrated.

The implications of the findings should also be more thoroughly explored. For example, if dynamic and static cues do not differ meaningfully, what does this imply for future cue-reactivity paradigms? Should alternative modalities (e.g., olfactory cues) be considered?

The discussion of limitations should be expanded. The restriction to non-obese participants is noted, but the relevance of this paradigm for populations such as individuals with obesity or binge eating disorder should be addressed in more detail.

Finally, the claim that the paradigm is “more naturalistic” is not sufficiently supported. While dynamic cues may increase activation in visual regions, no evidence is provided that they enhance ecologically valid cue reactivity. This conclusion should be more carefully qualified.

Reviewer #2: This is a timely and interesting pilot study addressing an important gap in the literature - namely, the lack of neuroimaging research comparing dynamic versus static food cues. The development of a dynamic stimulus paradigm is a clear strength, and the manuscript is generally well written and thoughtfully framed. That said, several methodological and interpretive issues limit the strength of the conclusions in its current form.

Major Comments

1. The analytic strategy would benefit from clearer separation between hypothesis-driven and exploratory approaches. Currently, ROIs are defined using a combination of anatomical masks and functional activation derived from the same dataset. This creates some ambiguity in the analytic framework. For a hypothesis-driven ROI analysis, it would be preferable to define ROIs independently (e.g., purely anatomical masks or regions defined from prior literature), extract the betas and perform the analyses. An alternative approach would be to combine all a priori masks into one mask (let's call it a reward mask) and then look at the effects within that reward mask and extract from the significant clusters within that mask.

Also, it would be helpful to show whole brain results rather than just depict them in a table.

2. High-calorie stimuli were always presented in Run 1 and low-calorie stimuli in Run 2. This fixed order introduces potential confounds (e.g., fatigue, habituation, satiety changes), making it difficult to interpret differences between high- and low-calorie conditions. This limitation should be more clearly acknowledged and discussed.

4.The use of a fixed 4-hour fasting period helps reduce variability in appetitive state; however, this approach may not fully standardize hunger across participants, given individual differences in metabolism and meal composition. It would strengthen the study to report (or control for) additional measures such as time since last meal, meal characteristics, or subjective hunger ratings. Without these measures, it is difficult to determine whether variability in appetitive state may have influenced the neural responses observed. This limits the ability to interpret the neural findings in terms of meaningful differences in appetite or motivation. Please expand the discussion of this limitation.

5. The manuscript currently suggests that dynamic cues do not “robustly amplify” reward-related activation. Given the limited power, this conclusion should be softened. The data are inconclusive rather than evidence of no effect.

6. Given that the dynamic stimulus set is a central contribution of this work, I encourage the authors to consider making the video stimuli publicly available, for example as supplementary materials or via a Github repository. This would substantially enhance reproducibility and facilitate reuse of the paradigm by other researchers.

Minor Comments

Please report behavioral results (e.g., appeal ratings) more fully rather than “data not shown.”

Consider reporting effect sizes (e.g., Cohen’s d) alongside p-values.

**********

what does this mean?). If published, this will include your full peer review and any attached files.

If you choose “no”, your identity will remain anonymous but your review may still be made public.

**Do you want your identity to be public for this peer review?**  For information about this choice, including consent withdrawal, please see our Privacy Policy

Reviewer #1: No

Reviewer #2: No

**********

[NOTE: If reviewer comments were submitted as an attachment file, they will be attached to this email and accessible via the submission site. Please log into your account, locate the manuscript record, and check for the action link "View Attachments". If this link does not appear, there are no attachment files.]

To ensure your figures meet our technical requirements, please review our figure guidelines: https://journals.plos.org/plosone/s/figures

You may also use PLOS’s free figure tool, NAAS, to help you prepare publication quality figures: https://journals.plos.org/plosone/s/figures#loc-tools-for-figure-preparation.

NAAS will assess whether your figures meet our technical requirements by comparing each figure against our figure specifications.

# Author comment: Author response to Decision Letter 1

**DOI:** 10.1371/journal.pone.0361667.r002

15 May 2026

Response letter to the reviewers and editor is attached as "ReviewerComments_PLOSONE.docx"

Attachment Submitted filename: ReviewerComments_PLOSONE.docx

# Peer review: Decision Letter 1

**DOI:** 10.1371/journal.pone.0361667.r003

**Authors:** Yoko Hasegawa

24 Jun 2026

Dear Dr. Yeum,

Thank you for submitting your manuscript to PLOS ONE. After careful consideration, we feel that it has merit but does not fully meet PLOS ONE’s publication criteria as it currently stands. Therefore, we invite you to submit a revised version of the manuscript that addresses the points raised during the review process.

Please submit your revised manuscript by Aug 08 2026 11:59PM. If you will need more time than this to complete your revisions, please reply to this message or contact the journal office at plosone@plos.org. When you're ready to submit your revision, log on to https://www.editorialmanager.com/pone/ and select the 'Submissions Needing Revision' folder to locate your manuscript file.

- A letter that responds to each point raised by the academic editor and reviewer(s). You should upload this letter as a separate file labeled 'Response to Reviewers'.
- A marked-up copy of your manuscript that highlights changes made to the original version. You should upload this as a separate file labeled 'Revised Manuscript with Track Changes'.
- An unmarked version of your revised paper without tracked changes. You should upload this as a separate file labeled 'Manuscript'.

If you would like to make changes to your financial disclosure, please include your updated statement in your cover letter. Guidelines for resubmitting your figure files are available below the reviewer comments at the end of this letter.

If applicable, we recommend that you deposit your laboratory protocols in protocols.io to enhance the reproducibility of your results. Protocols.io assigns your protocol its own identifier (DOI) so that it can be cited independently in the future. For instructions see: https://journals.plos.org/plosone/s/submission-guidelines#loc-laboratory-protocols. Additionally, PLOS ONE offers an option for publishing peer-reviewed Lab Protocol articles, which describe protocols hosted on protocols.io. Read more information on sharing protocols at https://plos.org/protocols?utm_medium=editorial-email&utm_source=authorletters&utm_campaign=protocols.

As the corresponding author, your ORCID iD is verified in the submission system and will appear in the published article. PLOS supports the use of ORCID, and we encourage all coauthors to register for an ORCID iD and use it as well. Please encourage your coauthors to verify their ORCID iD within the submission system before final acceptance, as unverified ORCID iDs will not appear in the published article. *Only*  the individual author can complete the verification step; PLOS staff *cannot*  verify ORCID iDs on behalf of authors.

We look forward to receiving your revised manuscript.

Kind regards,

Yoko Hasegawa

Academic Editor

PLOS One

Journal Requirements:

1. If the reviewer comments include a recommendation to cite specific previously published works, please review and evaluate these publications to determine whether they are relevant and should be cited. There is no requirement to cite these works unless the editor has indicated otherwise.

2. Please review your reference list to ensure that it is complete and correct. If you have cited papers that have been retracted, please include the rationale for doing so in the manuscript text, or remove these references and replace them with relevant current references. Any changes to the reference list should be mentioned in the rebuttal letter that accompanies your revised manuscript. If you need to cite a retracted article, indicate the article’s retracted status in the References list and also include a citation and full reference for the retraction notice.

**Additional Editor Comments:**

Comment 1:

Reference 18 (Devoto F.) is incomplete — journal name, volume, pages, and DOI are all missing. Please provide the full citation.

Comment 2:

References 7 and 20 are identical (Mehta et al., 2012, AJCN), as are References 8 and 21 (Schur et al., 2009, Int J Obes). Please consolidate these duplicate citations and renumber all references accordingly.

Comment 3:

The functional ROI definition was based on food vs. object contrasts (p<0.05, uncorrected) to identify active voxels, after which dynamic vs. static format effects were tested within those same ROIs. Please clarify in the Methods or Discussion whether this approach introduces circularity (double-dipping), and if so, how the independence of the primary analysis was maintained.

Comment 4:

Exploratory weight-group comparisons were conducted across multiple ROIs and calorie conditions without correction for multiple comparisons. Although labeled "exploratory," the reported p-values (e.g., p=.020, p=.007) risk being overinterpreted. Please explicitly state in the Results and/or Discussion that these p-values are uncorrected and should be regarded as hypothesis-generating only.

[Note: HTML markup is below. Please do not edit.]

Reviewers' comments:

Reviewer's Responses to Questions

**Comments to the Author**

Reviewer #2: All comments have been addressed

Reviewer #3: (No Response)

**********

2. Is the manuscript technically sound, and do the data support the conclusions?

Reviewer #2: Partly

Reviewer #3: Yes

**********

3. Has the statistical analysis been performed appropriately and rigorously?

Reviewer #2: Yes

Reviewer #3: Yes

**********

4. Have the authors made all data underlying the findings in their manuscript fully available?

The PLOS Data policy

Reviewer #2: No

Reviewer #3: Yes

**********

5. Is the manuscript presented in an intelligible fashion and written in standard English?

Reviewer #2: Yes

Reviewer #3: Yes

**********

Reviewer #2: I appreciate the authors carefully revising the manuscript to sufficiently address my concerns and questions.

Reviewer #3: This revised manuscript by Yeum et al examines a pilot study of neural responses to dynamic vs. static food and object cues. Seventeen adults (8 healthy weight, 9 overweight) completed two fMRI runs showing dynamic and static images of high-calorie and low-calorie foods and matched object cues. Primary analyses examined if differences in stimulus format impacted neural response to food cues in reward- and appetite-related ROIs. Exploratory whole-brain analyses were conducted and exploratory weight group analyses were conducted. They did not find any effect of stimulus format on activation in the ROI analyses. Whole-brain analyses found greater activation for dynamic vs. static cues. Exploratory group analyses found greater activation for dynamic food cues in individuals who were overweight in the dorsal striatum.

Of note, I was not an initial reviewer for this study. This is an interesting study, as the use of dynamic cues is more ecologically valid than static cues and there is evidence from the addiction literature that these cues are more salient than static cues. However, the length of the dynamic cue presentation limits its ecological validity and may have impacted the results. Additional comments are listed below:

1. I agree with the overall premise that dynamic vs. static food cues should be investigated and that these responses may differ in individuals who are overweight compared to those who are not. However, if the study was designed to answer the latter question, it is incredibly underpowered, even as a pilot study, with less than 10 participants in each group. While this is presented as exploratory in the last paragraph, I think this should be emphasized more given the extremely limited power to answer the question presented as a gap in the introduction.

2. How was pre-scan fasting verified?

3. 2-seconds per stimulus for the dynamic cues is quite short. Were participants able to truly experience the dynamic nature of the cues in that time-frame?

4. Effect sizes should be provided for the weight group analyses.

5. While I agree that findings related to dynamic vs. static images for the ROI analyses should be interpreted with caution, the fact that effect sizes were so small and a large sample would be needed to detect effects indicates that the image state (at least for very short videos) is not a very important factor for studying reward/satiation responses to food cues in the brain.

**********

what does this mean?). If published, this will include your full peer review and any attached files.

If you choose “no”, your identity will remain anonymous but your review may still be made public.

**Do you want your identity to be public for this peer review?**  For information about this choice, including consent withdrawal, please see our Privacy Policy

Reviewer #2: No

Reviewer #3: No

**********

[NOTE: If reviewer comments were submitted as an attachment file, they will be attached to this email and accessible via the submission site. Please log into your account, locate the manuscript record, and check for the action link "View Attachments". If this link does not appear, there are no attachment files.]

To ensure your figures meet our technical requirements, please review our figure guidelines: https://journals.plos.org/plosone/s/figures

You may also use PLOS’s free figure tool, NAAS, to help you prepare publication quality figures: https://journals.plos.org/plosone/s/figures#loc-tools-for-figure-preparation.

NAAS will assess whether your figures meet our technical requirements by comparing each figure against our figure specifications.

# Author comment: Author response to Decision Letter 2

**DOI:** 10.1371/journal.pone.0361667.r004

2 Oct 2026

We have attached a Word document of all of our responses to reviewers' comments. Please see "ReviewerComments_PLOSONE.docx".

Attachment Submitted filename: ReviewerComments_PLOSONE_20260716.docx

# Editor report: Decision Letter 2

**DOI:** 10.1371/journal.pone.0361667.r005

**Authors:** Yoko Hasegawa

8 Oct 2026

Dear Dr. Yeum,

Thank you for submitting your manuscript to PLOS One. After careful consideration, we feel that it has merit but does not fully meet PLOS One’s publication criteria as it currently stands. Therefore, we invite you to submit a revised version of the manuscript that addresses the points raised during the review process.

Please submit your revised manuscript by Nov 22 2026 11:59PM. If you will need more time than this to complete your revisions, please reply to this message or contact the journal office at plosone@plos.org. When you're ready to submit your revision, log on to https://www.editorialmanager.com/pone/ and select the 'Submissions Needing Revision' folder to locate your manuscript file.

- A letter that responds to each point raised by the academic editor and reviewer(s). You should upload this letter as a separate file labeled 'Response to Reviewers'.
- A marked-up copy of your manuscript that highlights changes made to the original version. You should upload this as a separate file labeled 'Revised Manuscript with Track Changes'.
- An unmarked version of your revised paper without tracked changes. You should upload this as a separate file labeled 'Manuscript'.

If you would like to make changes to your financial disclosure, please include your updated statement in your cover letter. Guidelines for resubmitting your figure files are available below the reviewer comments at the end of this letter.

If applicable, we recommend that you deposit your laboratory protocols in protocols.io to enhance the reproducibility of your results. Protocols.io assigns your protocol its own identifier (DOI) so that it can be cited independently in the future. For instructions see: https://journals.plos.org/plosone/s/submission-guidelines#loc-laboratory-protocols. Additionally, PLOS One offers an option for publishing peer-reviewed Lab Protocol articles, which describe protocols hosted on protocols.io. Read more information on sharing protocols at https://plos.org/protocols?utm_medium=editorial-email&utm_source=authorletters&utm_campaign=protocols.

As the corresponding author, your ORCID iD is verified in the submission system and will appear in the published article. PLOS supports the use of ORCID, and we encourage all coauthors to register for an ORCID iD and use it as well. Please encourage your coauthors to verify their ORCID iD within the submission system before final acceptance, as unverified ORCID iDs will not appear in the published article. *Only*  the individual author can complete the verification step; PLOS staff *cannot*  verify ORCID iDs on behalf of authors.

We look forward to receiving your revised manuscript.

Kind regards,

Yoko Hasegawa

Academic Editor

PLOS One

Journal Requirements:

If the reviewer comments include a recommendation to cite specific previously published works, please review and evaluate these publications to determine whether they are relevant and should be cited. There is no requirement to cite these works unless the editor has indicated otherwise.

Please review your reference list to ensure that it is complete and correct. If you have cited papers that have been retracted, please include the rationale for doing so in the manuscript text, or remove these references and replace them with relevant current references. Any changes to the reference list should be mentioned in the rebuttal letter that accompanies your revised manuscript. If you need to cite a retracted article, indicate the article’s retracted status in the References list and also include a citation and full reference for the retraction notice.

Additional Editor Comments:

The authors have satisfactorily addressed the substantive concerns raised during review. I recommend acceptance, subject to the following three minor corrections in the final manuscript. No additional experiments or external peer review are required.

1.Clarify the whole-brain findings in the Abstract: Please distinguish the dynamic-versus-static food-specific contrast, relative to objects, from the food-versus-object contrasts. The former showed significant activation in the right occipital fusiform gyrus for high-calorie foods. Findings in frontal, insular, and cingulate regions should be attributed to their respective contrasts rather than presented collectively as dynamic-versus-static effects.

2. Correct and clarify the effect-size reporting: The high-calorie effect-size range reported at line 333 (d = 0.11–0.46) does not match Table 1, which includes values from −0.11 to 0.46. Please ensure consistency between the text and table, revise the description “negligible to small” to reflect the reported magnitudes, and specify how Cohen’s d was calculated for the paired comparisons.

3. Clarify the sample-size statement: For the statement that “40 participants or more” would be required, please provide the effect size, statistical test, significance level, and target power used in the calculation. Alternatively, remove the numerical estimate and retain a general statement that larger, adequately powered studies are needed.

[Note: HTML markup is below. Please do not edit.]

[NOTE: If reviewer comments were submitted as an attachment file, they will be attached to this email and accessible via the submission site. Please log into your account, locate the manuscript record, and check for the action link "View Attachments". If this link does not appear, there are no attachment files.]

To ensure your figures meet our technical requirements, please review our figure guidelines: https://journals.plos.org/plosone/s/figures

You may also use PLOS’s free figure tool, NAAS, to help you prepare publication quality figures: https://journals.plos.org/plosone/s/figures#loc-tools-for-figure-preparation.

NAAS will assess whether your figures meet our technical requirements by comparing each figure against our figure specifications.

# Author comment: Author response to Decision Letter 3

**DOI:** 10.1371/journal.pone.0361667.r006

9 Oct 2026

Please find attached our point-by-point response to each comment.

Attachment Submitted filename: ReviewerComments_PLOSONE_20261008.docx

# Editor report: Decision Letter 3

**DOI:** 10.1371/journal.pone.0361667.r007

**Authors:** Yoko Hasegawa

13 Oct 2026

Neural responses to dynamic versus static food cues: A pilot functional MRI study

PONE-D-26-09302R3

Dear Dr. Yeum,

We’re pleased to inform you that your manuscript has been judged scientifically suitable for publication and will be formally accepted for publication once it meets all outstanding technical requirements.

Within one week, you’ll receive an e-mail detailing the required amendments. When these have been addressed, you’ll receive a formal acceptance letter and your manuscript will be scheduled for publication.

An invoice will be generated when your article is formally accepted. Please note, if your institution has a publishing partnership with PLOS and your article meets the relevant criteria, all or part of your publication costs will be covered. Please make sure your user information is up-to-date by logging into Editorial Manager at Editorial Manager® and clicking the ‘Update My Information' link at the top of the page. For questions related to billing, please contact billing support.

If your institution or institutions have a press office, please notify them about your upcoming paper to help maximize its impact. If they’ll be preparing press materials, please inform our press team as soon as possible -- no later than 48 hours after receiving the formal acceptance. Your manuscript will remain under strict press embargo until 2 pm Eastern Time on the date of publication. For more information, please contact onepress@plos.org.

Kind regards,

Yoko Hasegawa

Academic Editor

PLOS One

Additional Editor Comments (optional):

Reviewers' comments:

# Editor report: Acceptance letter

**DOI:** 10.1371/journal.pone.0361667.r008

**Authors:** Yoko Hasegawa

PONE-D-26-09302R3

PLOS One

Dear Dr. Yeum,

I'm pleased to inform you that your manuscript has been deemed suitable for publication in PLOS One. Congratulations! Your manuscript is now being handed over to our production team.

At this stage, our production department will prepare your paper for publication. This includes ensuring the following:

* All references, tables, and figures are properly cited

* All relevant supporting information is included in the manuscript submission,

* There are no issues that prevent the paper from being properly typeset

You will receive further instructions from the production team, including instructions on how to review your proof when it is ready. Please keep in mind that we are working through a large volume of accepted articles, so please give us a few days to review your paper and let you know the next and final steps.

Lastly, if your institution or institutions have a press office, please let them know about your upcoming paper now to help maximize its impact. If they'll be preparing press materials, please inform our press team within the next 48 hours. Your manuscript will remain under strict press embargo until 2 pm Eastern Time on the date of publication. For more information, please contact onepress@plos.org.

You will receive an invoice from PLOS for your publication fee after your manuscript has reached the completed accept phase. If you receive an email requesting payment before acceptance or for any other service, this may be a phishing scheme. Learn how to identify phishing emails and protect your accounts at https://explore.plos.org/phishing.

If we can help with anything else, please email us at customercare@plos.org.

Thank you for submitting your work to PLOS One and supporting open access.

Kind regards,

PLOS One Editorial Office Staff

on behalf of

Dr. Yoko Hasegawa

Academic Editor

PLOS One
